# DNMT3B supports meso-endoderm differentiation from mouse embryonic stem cells

**DOI:** 10.1038/s41467-023-35938-x

**Published:** 2023-01-23

**Authors:** Andrea Lauria, Guohua Meng, Valentina Proserpio, Stefania Rapelli, Mara Maldotti, Isabelle Laurence Polignano, Francesca Anselmi, Danny Incarnato, Anna Krepelova, Daniela Donna, Chiara Levra Levron, Giacomo Donati, Ivan Molineris, Francesco Neri, Salvatore Oliviero

**Affiliations:** 1 Department of Life Sciences and Systems Biology & Molecular Biotechnology Center - MBC, Università di Torino, Via Nizza 52, 10126 Torino, Italy; 2 Italian Institute for Genomic Medicine (IIGM), Sp142 Km 3.95, 10060 Candiolo, Torino Italy; 3 Department of Molecular Genetics, Groningen Biomolecular Sciences and Biotechnology Institute (GBB), University of Groningen, Groningen, the Netherlands

**Keywords:** Differentiation, Epigenetic memory, Embryonic stem cells

## Abstract

The correct establishment of DNA methylation patterns during mouse early development is essential for cell fate specification. However, the molecular targets as well as the mechanisms that determine the specificity of the de novo methylation machinery during differentiation are not completely elucidated. Here we show that the DNMT3B-dependent DNA methylation of key developmental regulatory regions at epiblast-like cells (EpiLCs) provides an epigenetic priming that ensures flawless commitment at later stages. Using in vitro stem cell differentiation and loss of function experiments combined with high-throughput genome-wide bisulfite-, bulk-, and single cell RNA-sequencing we dissected the specific role of DNMT3B in cell fate. We identify DNMT3B-dependent regulatory elements on the genome which, in *Dnmt3b* knockout (3BKO), impair the differentiation into meso-endodermal (ME) progenitors and redirect EpiLCs towards the neuro-ectodermal lineages. Moreover, ectopic expression of DNMT3B in 3BKO re-establishes the DNA methylation of the master regulator Sox2 super-enhancer, downmodulates its expression, and restores the expression of ME markers. Taken together, our data reveal that DNMT3B-dependent methylation at the epiblast stage is essential for the priming of the meso-endodermal lineages and provide functional characterization of the de novo DNMTs during EpiLCs lineage determination.

## Abstract (web summary)

DNMT3B-dependent DNA methylation provides epigenetic priming at epiblast stage by methylating enhancers of ectodermal genes to ensure proper differentiation toward mesoendoderm tissues.

## Introduction

In mammals, the epigenome is extensively remodelled during early stages of development^1^. DNA methylation (DNAme) of cytosine residues, which introduces 5-methylcytosine (5mC), mainly at CpGs, controls the DNA binding of transcription factors^2^ and plays a critical role in gene regulation. It is established and stably propagated during cell specification. DNAme is introduced by the de novo DNMT3A and DNMT3B DNA methyltransferases together with DNMT3L, while DNMT1 together with UHRF1 are mainly involved in the propagation of the DNAme on the genome during DNA replication^3,4^.

In the early embryo global DNA hypomethylation erases the epigenetic memory^5^. The exit from pluripotency is characterized by the transition from the pre- to the post-implantation epiblast of the embryo. During this transition genome-wide DNAme is established by the de novo DNMT3A and DNMT3B DNA methyltransferases which are strongly upregulated to establish the DNAme essential for cell fate specification during development and transcription integrity^6–11^.

In mice *Dnmt3a* knockout results in postnatal lethality, while *Dnmt3b* knockout results in embryonic lethality indicating distinct biological functions of the de novo DNMTs^12,13^. Biochemical and structural evidence indicate that DNMT3A and DNMT3B exhibit preferences for specific flanking sequences^14^. In mouse embryonic stem cells (ESCs) DNMT3A has been shown to mainly methylate shores of bivalent CpG island^15,16^, while DNMT3B preferentially binds to the gene body of active genes^17,18^. However, the specific targets of the de novo DNMTs involved in cell fate specification are not yet clarified.

ESCs and epiblast-like cells (EpiLCs) represent respectively the naive and prime state of pluripotency corresponding to pre- and post-implantation epiblast of the embryo. ESCs are characterized by low levels of de novo DNMTs, high levels of DNMT3L and TET1 and TET2, high DNAme turnover and general hypomethylation, while EpiLCs express high levels of de novo DNMTs and dramatic increase of DNAme^19–29^. To examine the DNAme specific role in somatic cell specification we investigated the developmental trajectories of *Dnmt* knockout ESCs along their exit from pluripotency. By whole genome DNAme coupled with gene expression and cell phenotype analysis we here demonstrate a specific role of DNMT3B, but not DNMT3A, in the meso-endoderm (ME) lineage specification and identified DNMT3B genomic targets that prime EpiLCs toward ME.

## Results

### Knockout of *Dnmt3b* in ESCs impairs embryoid body differentiation

**Fig. 1:**
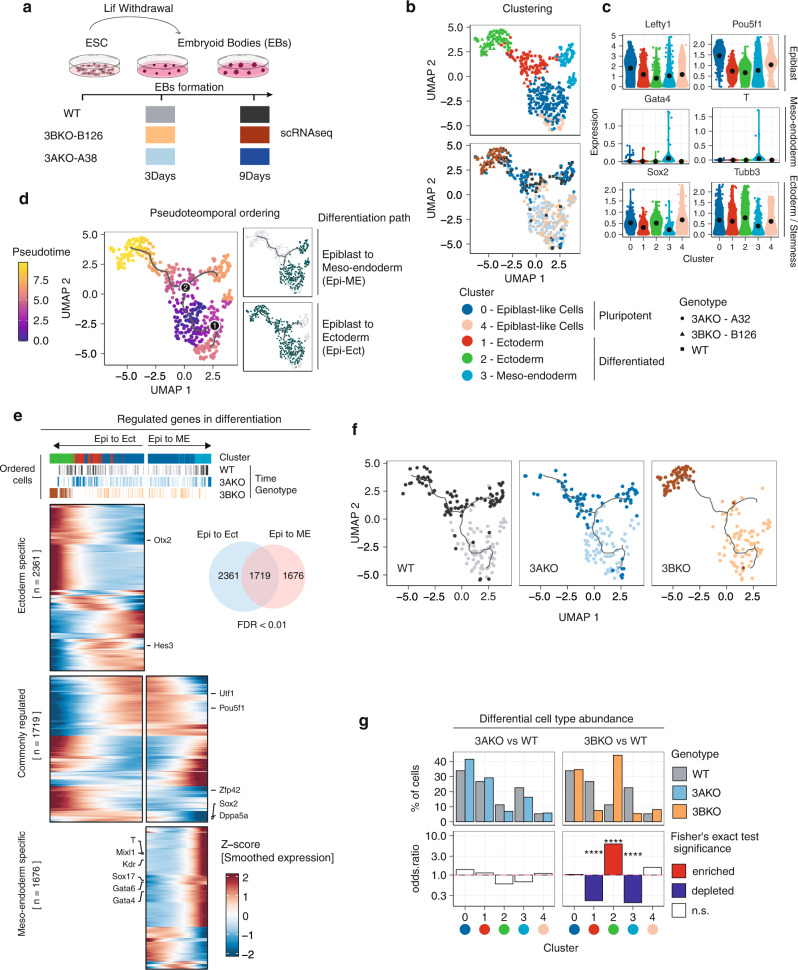
scRNA-seq profiling of *Dnmt3a*^*−/−*^ and *Dnmt3b*^*−/−*^ differentiating EBs. **a** Overview of the experimental design and visualisation of collected time points. The colour scheme for WT, 3AKO and 3BKO samples will be kept throughout the manuscript. **b** UMAP embedding of 487 WT, 3AKO and 3BKO single cell transcriptomes. Cells are coloured by cluster (top panel) and genotype/time of cells’ collection (bottom panel). 3AKO = lightblue/darkblue, 3BKO = light orange (3Days)/dark orange (9Days), WT = light grey (3Days)/dark grey (9Days). **c** Gene expression levels distribution of representative epiblast (*Lefty1*, *Pou5f1*), meso-endoderm (*Gata4*, *T*) and ectoderm markers (*Sox2*, *Tubb3*) in the five identified cell clusters. **d** Pseudotime analysis of single cell differentiation trajectories with reverse graph embedding^33^. The line plot on the UMAP represents the embedded trajectory graph. Cells are coloured according to pseudotime (left panel) and differentiation paths (i.e. Epi-ME, Epi-Ect, right panels). **e** UMAP visualisation of the reconstructed differentiation trajectories for each source cell type (i.e. WT, 3AKO and 3BKO genotype). **f** Heatmap showing the expression patterns of genes differentially regulated in pseudotime along the two differentiation branches, performed via the graph-autocorrelation analysis method^33^. Genes are grouped according to the branch in which they show significant variation (FDR < 0.01): Epi-to-Ect specific (top cluster), Epi-to-ME (bottom cluster) specific or regulated in both branches (mid cluster). **g** Barplots showing the differential cell type abundance in terms of the genotype of origin (i.e. WT, 3AKO and 3BKO) in each of the five identified cell clusters. For each cluster, the relative proportion of mutant cells (3AKO, 3BKO) was compared with WT cells using Fisher’s exact test. Top panels report the percentage of cells in each cluster for the indicated comparisons (i.e. 3AKO vs WT, 3BKO vs WT). Bottom panels report the odds ratio from Fisher’s exact test, coloured for their significance (enriched = red, depleted = blue, non-significant = white, ****p value < 0.0001, one-sided).

To investigate the impact of the loss of de novo DNA methyltransferases in vitro during the early stages of development, we leveraged the *Dnmt3a* and *Dnmt3b* homozygous knockouts we have previously generated in E14 mouse embryonic stem cell (ESCs)^18,29^. Wild type (WT) and two independent *Dnmt3a*^−/−^ (3AKO), and *Dnmt3b*^−/−^ (3BKO) ESC clones were differentiated into three-dimensional embryoid body structures (EBs) by LIF withdrawal (Fig. 1a and Supplementary Fig. 1). In the absence of LIF and employing ultra-low attachment plates, cultured cells aggregate spontaneously aggregate and form 3D structures over a 9-day time course. EBs represent a simple and useful model to mimic the differentiation of ESCs into the three germ lineages, under controlled in vitro conditions^25,30^.

To analyse the gene expression patterns of the EBs at the resolution of individual cells, we collected samples at 3 and 9 days during EBs differentiation from WT and mutant cells (Fig. 1a) and profiled their gene expression by single cell RNA-sequencing as in ref. ^31^.

We performed unsupervised clustering using the Louvain algorithm from the Seurat pipeline^32^, and visualised the results by Uniform Manifold Approximation and Projection (UMAP) embedding. This analysis divided the cells into five main clusters (Fig. 1b), that we annotated according to the expression of known markers of early embryonic cell populations (Fig. 1c and Supplementary Data 1). In particular, we labelled cluster 0 and 4 as ‘pluripotent Epiblast-like cells (Epi)’, as they presented high expression of genes like *Lefty1* and *Pou5f1* (Fig. 1c); cluster 1 and 2 as ‘ectoderm (Ect)’, as defined by the expression of ectodermal markers such *Tubb3* and *Sox2* (Fig. 1c); cluster 3 as ‘meso-endoderm (ME)’, as they expressed genes like *T* and *Gata4* (Fig. 1c).

Looking at the distribution of the different clones (i.e. 3AKO, 3BKO, and WT) into the UMAP (Fig. 1b, lower panel), we observed that, while at day 3 of differentiation the three populations were uniformly distributed among clusters, at day 9 a clear separation emerged between 3AKO and 3BKO cells. Combining the clustering results with the pseudo-temporal ordering of cell trajectories, performed with Monocle3^33^, we defined two main differentiation paths, connecting the two physical-time points of samples collection: one going from Epiblast-like cells at day 3 to ectoderm at day 9, the other going from Epiblast-like cells to ME at day 9 (Fig. 1d). The analysis of differentially regulated genes in pseudotime along these paths (FDR < 0.01, performed via the graph-autocorrelation analysis, Supplementary Data 2), revealed three distinct groups (Fig. 1e): Epi-to-Ect specific, Epi-to-ME specific, and commonly regulated genes in both branches. Among the genes upregulated on the Epi-to-ME path, we found mesodermal (i.e. *T*, *Mixl1* and *Kdr*) and endodermal markers (i.e. *Gata4*, *Gata6*, and *Sox17*). In Epi-to-Ect path, we observed upregulation of early ectodermal markers (i.e. *Otx2* and *Hes3*) as well as sustained expression of genes already expressed in the pluripotent stage (i.e. *Sox2*), which were downregulated in the Epi-to-ME branch (Fig. 1e). Interestingly, while WT and 3AKO cells contained comparable proportions of cells in all clusters (Fig. 1f, g), 3BKO cells were significantly depleted in the ME cluster and were enriched in the ectodermal clusters (Fig. 1f, g, Supplementary Fig. 1 and Supplementary Table 1). These results suggest that, while 3AKO cells display the same differentiation potential of WT cells towards both the ectodermal and ME paths, 3BKO cells are significantly impaired in their ME commitment.

### Loss of DNMT3B does not affect the formation of EpiLCs, but impairs meso-endoderm and favours ectoderm differentiation

**Fig. 2:**
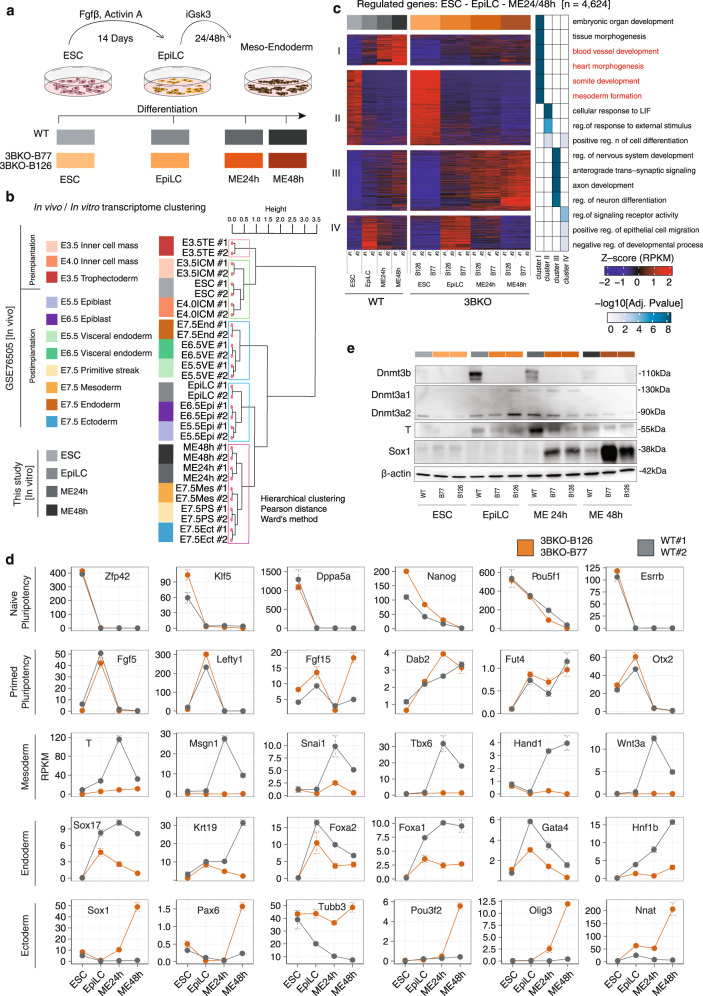
Loss of DNMT3B impairs meso-endoderm lineage commitment. **a** Schematic representation of the two-step differentiation model from ESCs to EpiLCs first with Fgfβ and Activin A, and then to meso-endoderm (ME) progenitors with iGsk3. The time points of cells’ collection are reported in the colour-code used throughout the figures (i.e., shades of grey for WT, shades of orange for 3BKO). **b** Hierarchical clustering of RNA-seq data from the in vitro differentiation and in vivo embryonic tissues derived from pre- and post-implantation mouse embryos^35^. Pearson’s correlation distance and Ward’s method were employed to perform the analysis. **c** On the left, an RNA-seq heatmap showing the results of gene expression profiles clustering with K-means for WT and 3BKO (two independent clones) cells during the complete differentiation time course (ESC-EpiLC-ME). DEGs arising during the differentiation time course in any group were identified by ANOVA-like test with edgeR^54^. Rows are genes, columns are samples and the scaled expression level (Z-score RPKM) is plotted. On the right, heatmap showing selected GO terms for enriched biological processes in each cluster. Terms related to meso-endoderm are highlighted in red. **d** Gene expression time-course for stage-specific pluripotency (naive, primed) and germ layers (mesoderm, endoderm, ectoderm) marker genes. Dots represent normalized RPKM values, averaged by replicates/conditions (*n* = 2 biological replicates for each genotype or clone at each time point). Error bars represent standard errors. **e**, Western blot analysis of the de novo DNMTs (Dnmt3a1, Dnmt3a2, Dnmt3b), T (mesodermal marker) and Sox1 (neuro-ectodermal marker) expression during the differentiation time course. β-actin serves as loading control. Representative of two independent experiments. Uncropped gels are provided in Supplementary Fig. 11.

To gain insights into the functional role of DNMT3B-dependent DNAme in ME differentiation, we switched to a two-step ME-specific in vitro differentiation model (Fig. 2a). To mimic the passage through primed pluripotency in the early post-implantation embryo stage (E5.5) and the subsequent specification of the primitive streak, we first differentiated ESCs into epiblast-like stem cells (EpiLCs) via induction of the activin/nodal pathway for 14 days, followed by a faster conversion of EpiLCs towards ME through inhibition of Gsk3b (and concomitant activation of WNT)^34^.

We investigated the effects of *Dnmt3b* loss during ME specification by performing gene expression profiling using RNA sequencing (RNA-seq) on WT and two independent 3BKO clones (Fig. 2a). The level of DNMT3A, DNMT3L, and TET enzymes were unchanged in 3BKO with only a modest increase of DNMT1 (Supplementary Fig. 2a).

Comparative analysis by unsupervised hierarchical clustering of the in vitro differentiation with published RNA-seq of in vivo embryonic tissues^35^ correctly clustered ESCs together with pre-implantation Inner cell Mass (ICM), EpiLCs cells together with E5.5 and E6.5 EpiLCs, and 24 hours (h) and 48 h ME cells together with E7.5 Mesoderm and Endoderm (Fig. 2b). Thus, the in vitro differentiation largely recapitulates the transcriptomic changes that occur in vivo.

Differential expression analysis revealed 4624 differentially expressed genes (DEGs) arising over the time course, that were grouped into four gene clusters with distinct patterns of gene expression dynamics in WT and 3BKO cells (Fig.2c and Supplementary Data 3, 4). Clusters II and IV showed similar expression patterns in WT and 3BKO cells. In particular, Cluster II was characterised by genes that are downregulated after the exit from the ESC-pluripotent state and was enriched for genes associated with pluripotency, including naïve pluripotency markers such as *Nanog*, *Esrrb*, *Dppa3a* and *Zfp42*. Cluster IV included genes upregulated at the exit from the ESC-pluripotent state and was enriched for genes associated with the epiblast stage, including primed pluripotency markers such as *Fgf5*, *Lefty1*, and *Otx2*^36^ (Supplementary Fig. 2b, c). In contrast, clusters I and III were characterised by genes differently expressed between WT and 3BKO cells. Specifically, cluster I was composed of genes upregulated in WT cells after ME commitment, including *T*, *Msgn1*, *Snai1*, *Hand1*, *Sox17*, *Krt19*, *Foxa1*, which were downregulated in 3BKO cells, while cluster III included genes upregulated in 3BKO and was enriched for neuronal and ectodermal genes such as *Sox1*, *Pax6*, *Tubb3* and *Pou3f2* (Fig. 2c, d and Supplementary Data 3, 4).

Also, at the protein level, Western blot and immunostaining analyses confirmed that T starts to be expressed at the EpiLCs stage and, in the WT, it peaks 24 h after the induction of ME differentiation, but has a visible reduction in 3BKO cells. Conversely, the neuroectodermal marker SOX1 is induced only in the 3BKO from 24 h with a further strong induction at 48 h (Fig. 2e and Supplementary Fig. 2d, e). 3BKO did not alter cell proliferation (Supplementary Fig. 2f) nor the expression of cell cycle and apoptosis genes (Supplementary Fig. 2g).

**Fig. 3:**
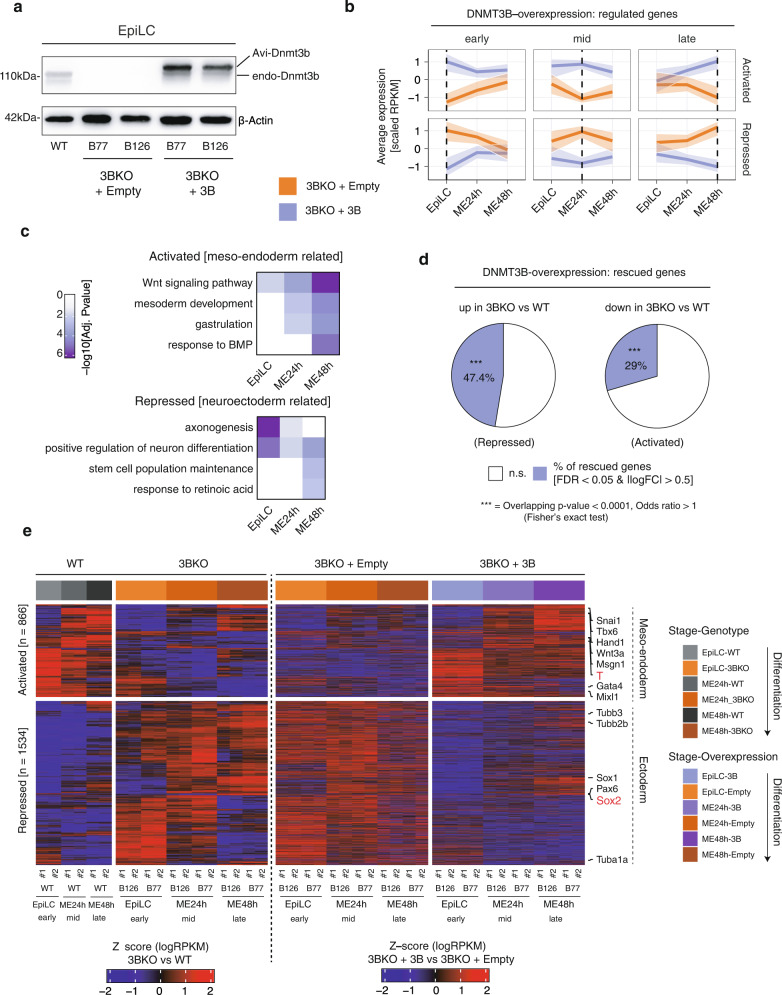
Rescue of the meso-endoderm phenotype in *Dnmt3b*^*−/−*^ cells. **a** Western blot analysis of DNMT3B expression in 3BKO (two independent clones) EpiLC derived from ESCs transfected with Empty vector (control) or Avitag-Dnmt3b (Rescued). WT cells and β-actin serve as control. Representative of two independent experiments. Uncropped gels are provided in Supplementary Fig. 11. **b** Trends of gene expression dynamics for all the DEGs (activated at the top, repressed at the bottom) between 3BKO + empty (3BKO) (orange) and 3BKO + 3B (rescued) (purple) during differentiation from EpiLC to ME24 and ME48h. Genes are grouped as early, medium and late according to when the biggest difference in transcription between 3BKO + 3B (rescued) and 3BKO + empty (3BKO) occurs. Dark lines represent the median, the shaded areas represent the interquartile range of each gene set. **c** Heatmap showing selected GO terms for enriched biological processes in activated and repressed genes, grouped as in **b**. **d** Pie chart showing the proportion of rescued genes by DNMT3B ectopic expression in 3BKO cells (***p-value < 0.0001, Odds ratio > 1, one-sided Fisher’s exact test). **e** Heatmaps showing the expression patterns of rescued genes from panel **d**.

We performed DNMT3B rescue experiments of both 3BKO ESC clones by DNMT3B ectopic expression using an empty vector (Empty) as a negative control (Fig. 3a) and performed RNA-seq during the differentiation from EpiLCs to ME. We analysed DEGs at different time points between 3BKO and DNMT3B rescued cells (Fig. 3b and Supplementary Data 5). GO enrichment analysis highlighted that ME-related gene sets, including WNT signalling pathway, mesoderm development and response to BMP, were upregulated in DNMT3B rescue. Conversely, gene sets related to neuroectoderm, as axonogenesis and positive regulation of neuronal differentiation, were repressed (Fig. 3c). We observed that 47.4% of the upregulated genes in the 3BKO were repressed and 29% were reactivated by DNMT3B ectopic expression (Fig. 3d, e). In particular, among the induced TFs, we found *T*, *Gata4*, and *Mixl1*, known to be involved in the ME commitment, while *Sox2*, *Pax6*, and *Tubb3*, which are markers of neuro-ectoderm specification, were significantly repressed upon DNMT3B ectopic expression (Fig. 3e and Supplementary Data 5).

Taken together, these results demonstrate that lack of DNMT3B does not affect the formation of EpiLCs from ESCs, but impairs their further differentiation into ME redirecting the differentiation of EpiLCs towards the neuro-ectodermal transcriptional program.

### Lack of DNMT3B impairs DNAme in EpiLCs at critical regulatory regions for their commitment toward meso-endoderm

To obtain a detailed map of the DNAme landscape established during the ESC-EpiLC-ME differentiation, we interrogated WT and two different 3BKO clones at the ESC, EpiLC and ME stages by whole genome bisulfite sequencing (WGBS). PCA and hierarchical clustering analysis showed good overall correlation between biological replicates and well-discriminated consecutive differentiation steps (Supplementary Fig. 3a–c). Globally, we observed a gradual increase of DNAme levels from ESCs to ME48h, with reduced DNAme in the 3BKO samples, both in terms of average DNAme and percentage of highly methylated CpG sites (Supplementary Fig. 3d, e).

To verify that the in vitro data genuinely describe what happens in vivo, we compared the WGBS at EpiLCs with published data of E6.5 epiblast derived from WT and 3BKO embryos^37^. We found that 40.3% of the in vitro DMRs were also hypomethylated in vivo, with significant enrichment in distal enhancers associated with genes involved in positive regulation of neuron differentiation and gastrulation (Supplementary Fig. 4). Interestingly, we also observed that a large fraction of the genes having an hypomethylated region in human 3BKO HUES64^38^ were consistently hypomethylated in EpiLCs with enrichment for enhancers of genes involved in neural differentiation (Supplementary Fig. 5).

We next defined differentially methylated regions (DMRs) in WT cells differentiation from ESCs to ME48h, resulting in a total of ~54,000 regions (Supplementary Fig. 6a and Supplementary Data 6). These DMRs cluster into three distinct groups, defined as medium (i), high (ii) and demethylated (III) according to the trend and magnitude of the overall DNAme levels acquired. Genomic annotation of these three DMR clusters showed significant enrichment in regulatory regions (Supplementary Fig. 6b, c) of genes involved in development (cluster I and III) and regulation of metabolism (cluster II).

**Fig. 4:**
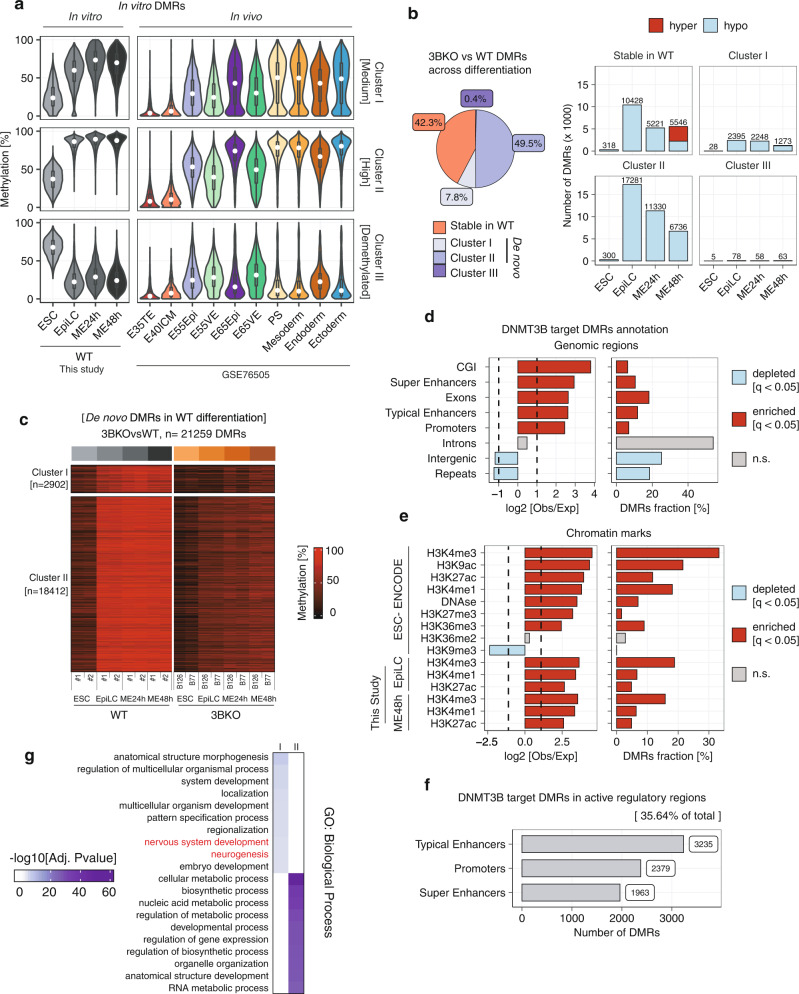
WGBS analysis identifies DNMT3B-dependent de novo methylated regions during ESC-EpiLC-ME differentiation. **a** Violin plots comparing the behaviour of DNAme levels (as average %) at the DMRs identified in WT differentiation (see Supplementary Fig. 6) between in vitro differentiation and in vivo embryonic and extraembryonic tissues from published data^35^. White dots indicate median, box indicates the interquartile range (IQR) and whiskers denote the 1.5 × IQR. **b** (left) Pie chart of proportions of DMRs identified during the ESC-EpiLC-ME differentiation in 3BKO cells, divided as “stable in WT” or belonging to WT differentiation cluster I, II or III. (right) Barplots showing the number of 3BKO versus WT DMRs in each group, coloured according to hypo- (pale blue) or hyper- (red) methylation. **c** WGBS heatmap visualising the DNAme dynamics across differentiation between 3BKO and WT for the two DMR clusters (cluster I and II) that are targets of DNMT3B for de novo DNAme. **d**, **e** Annotation of DMRs for the overlap with distinct genomic regions (**d**) and chromatin marks (**e**), reported as (left) the log2-enrichment for each feature and (right) the percentage of DMRs overlapping each feature, calculated with the Genomic Association Test (GAT) tool^39^. Bars are coloured according to the statistical significance (*q*-value < 0.05) of each feature (enriched = red, depleted = pale blue, non-significant = grey). **f** Barplot showing the number of DNMT3B target DMRs overlapping regulatory regions (promoters, typical and super enhancers). Typical and super enhancers were defined by the H3K27ac signal profiles across differentiation using ROSE^60^ (Supplementary Fig. 8). **g** Heatmap showing adjusted *p*-values for selected GO terms fof enriched biological processes in each cluster. Gene set over-representation analysis was performed for genes associated with DMRs overlapping putative regulatory regions using hypergeometric tests as implemented in GREAT^63^.

The DNAme patterns of each of the three DMR clusters largely mirror the trend of in vivo DNAme data from peri- and post implantation mouse embryos from E3.5 to E7.5^35^ (Fig. 4a and Supplementary Fig. 6e–j). Detailed analysis showed that the largest fraction of DMRs exhibit the same dynamics and DNAme levels between in vitro and in vivo (54.8% of cluster I which includes 5528 regions and 77% of cluster II which includes 46982 regions) (Supplementary Fig. 6a, e, h and Supplementary Fig. 7). We also observed a fraction of DMRs that reach lower levels of DNAme in vivo as compared to in vitro (19.8% of cluster I and 21% of cluster II), and a fraction of genomic regions that acquire de novo DNAme only in the in vitro system (25.4% of cluster I and 2% of cluster II), remaining mostly hypomethylated in vivo.

The comparison of the DNAme dynamics across ME differentiation between 3BKO and WT samples showed that 42.3% regions did not change their DNAme state during WT differentiation (Fig. 4b), so we defined them as ‘stable in WT’. The remaining regions overlapped the three WT DMR clusters (Fig. 4a and Supplementary Fig. 6a). We focused our attention on cluster I and II as these corresponded to regions that were de novo methylated in WT cells that failed to acquire DNAme in 3BKO cells (Fig. 4c), thus being DNMT3B-target regions (DNMT3B DMRs) for de novo DNAme during the differentiation process. Interestingly, both cluster I (Medium)—which accounts for 7.8% of the identified DMRs—and cluster II (high)—which comprises 49.3% of total DMRs—are made of regions that gained DNAme during the transition from ESCs to EpiLCs and maintain their DNAme status afterwards.

The genomic distribution of DNMT3B DMRs showed a significant over-representation at CpG Islands (6.3%), promoters (6.9%) and exons (18.2%), as well as at enhancers and super enhancers regions (Fig. 4d, *Q*-value < 1e^−4^ as calculated by the GAT tool^39^). In particular, we found that 7577 DNMT3B-DMRs (35.64% of total) overlapped with regulatory regions, either promoters or enhancers that were active (i.e. marked by H3K27ac) in any of the three stages of the differentiation (ESCs, EpiLCs or ME) (Fig. 4e and Supplementary Fig. 8), with a strong enrichment at both typical- and super-enhancer (respectively of 11.8% and 10.5% of nucleotide overlap, log2[Obs./Exp.] ratio > 2 and *Q*-value < 1e^−4^) elements (Fig. 4e, f). Functional enrichment analysis of genes associated with each DMR cluster revealed a significant enrichment for GO terms related to development (Fig. 4g).

**Fig. 5:**
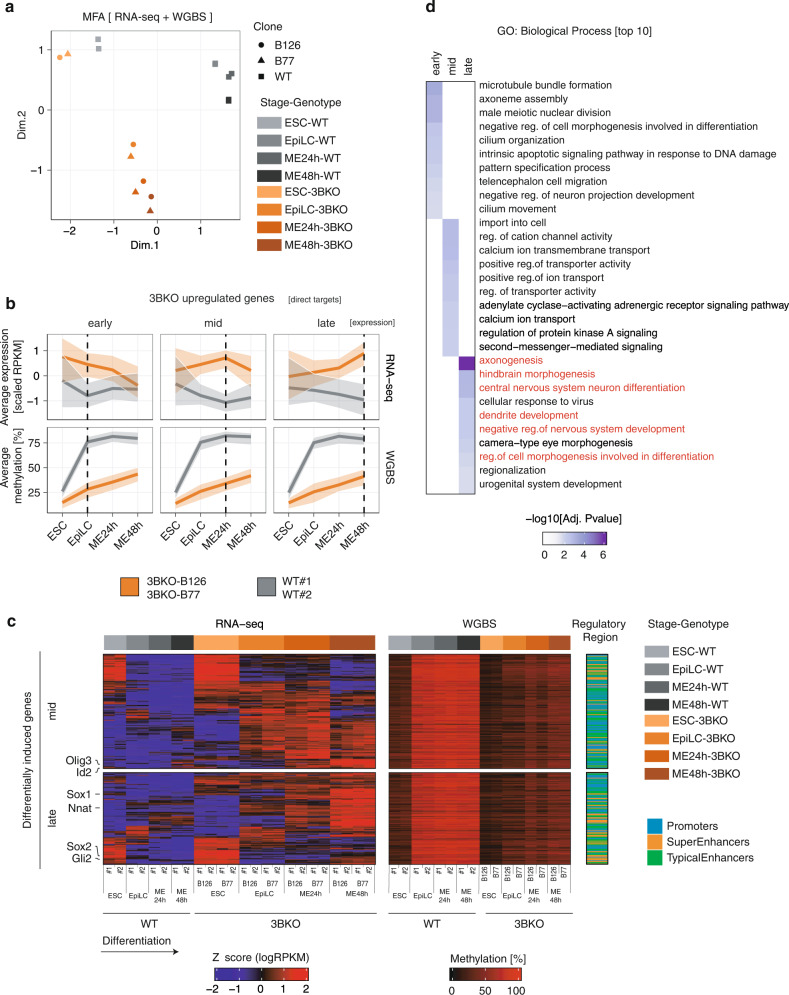
Integrated analysis reveals key DNMT3B regulated genes associated with neuro-ectodermal lineage commitment. **a** Multiple Factor Analysis (MFA) approach on integrated RNA and WGBS sequencing data show discrimination between ESC, EpiLC and ME stages and a divergent differentiation trajectory between 3BKO and WT cells. **b** Trends of gene expression and DNAme dynamics in WT (grey) and 3BKO (orange) cells for the 615 DNMT3B target genes. Dark lines represent median of gene expression (top panel, as scaled RPKM) and DMRs methylation (bottom panel, as %), shaded areas represent the interquartile range. Genes are divided as early, medium and late according to when the biggest difference in transcription between 3BKO and WT occurs (indicated by the vertical line). While transcription of these genes shows distinct dynamical patterns of induction, DNAme depositions on their associated regulatory region is mostly established at the EpiLC stage. **c**, Integrated heatmaps of RNA-seq (left) and WGBS data (right) of differentially induced genes in medium and late stages of differentiation, displaying also differential methylation in regulatory regions (promoters/enhancers/super-enhancers). Each row represents a z-score of normalized logRPKM (for RNA-seq) and average % of DNAme (for WGBS) of the associated DMRs. The last (rightmost) 1-column heatmap indicates the regulatory region. Key neuro-ectodermal markers are indicated on the left. **d** Heatmap showing adjusted *p*-values (hypergeometric test as implemented in the ClusterProfiler package) of GO terms for enriched biological processes in early, medium and late DNMT3B target genes.

We then integrated the results from the joint profiling of gene expression (RNA-seq), DNAme (WGBS) and histone modifications associated with active or primed regulatory regions (ChIP-seq of H3K4me1/me3, H3K27ac) during ME induction in 3BKO in comparison to WT cells (Fig. 5).

Multiple Factor Analysis (MFA) applied on the integrated RNA-seq and WGBS datasets confirmed the divergent differentiation trajectories of WT and 3BKO cells (Fig. 5a). By crossing these multi-omics profiles, we identified a list of 615 DNMT3B target genes, defined as: (i) having at least one associated DMR targeted by DNMT3B across the differentiation time course (WGBS); (ii) overlapping with a putative regulatory region (ChIP-seq); (iii) being upregulated in 3BKO cells with respect to WT upon ME induction (RNA-seq).

We next visualised the gene expression and DNAme dynamics of the hypomethylated genes. Interestingly, while the gene upregulation in 3BKO samples followed distinct patterns, peaking at either the EpiLCs, ME24h or ME48h stages, the difference in the DNAme level of their corresponding regulatory regions showed a unique behaviour, with DNAme deposition at EpiLCs in WT cells (Fig. 5b). This DNMT3B-dependent DNAme is stable over ME differentiation and the delta between WT and 3BKO persists throughout ME with 3BKO DNAme never reaching the WT level. Thus DNMT3B-dependent DNAme primes EpiLCs toward ME.

Heatmap of the mid and late 3BKO upregulated genes and gene set enrichment analysis of all these groups (Fig. 5c, d) revealed a significant enrichment for genes involved in neuro-ectodermal lineage commitment, including key markers such as *Sox1, Sox2, Nnat, Gli2*, and *Olig3* (Fig. 5c, Supplementary Fig. 9 and Supplementary Data 7).

These results indicate that the loss of DNMT3B-dependent DNAme at the primed pluripotency stage is directly responsible for impairing the differentiation into ME, in favour of the neuro-ectodermal transcriptional program.

### DNMT3B-dependent DNAme of the Sox2 super enhancer in EpiLCs is required for meso-endodermal specification

**Fig. 6:**
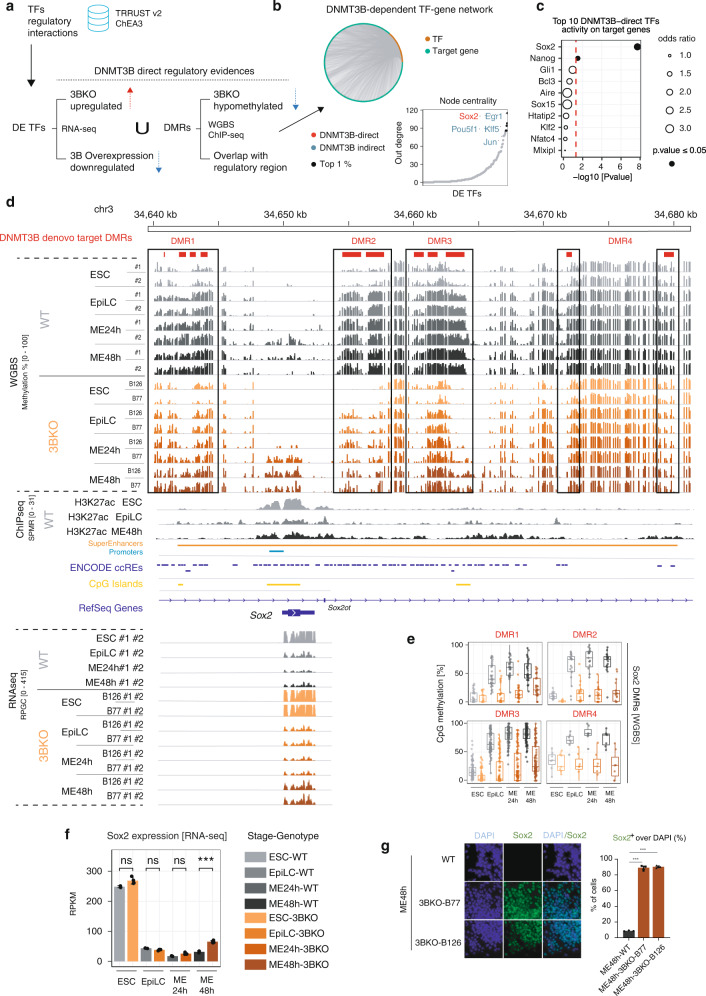
DNMT3B-dependent DNA methylation regulates the super-enhancer of *Sox2* neuro-ectodermal master regulator. **a** Schematic of the workflow for the DNMT3B-dependent regulatory network reconstruction. Starting from the integrated TF-target regulatory interactions retrieved from TRRUSTv2^40^ and ChEA3^41^ databases, the network was filtered for DEGs and the DNMT3B-direct regulatory evidence was used to classify nodes as direct (i.e. upregulation in 3BKO, downregulation in DNMT3B overexpression, association to at least one 3BKO-hypomethylated DMR overlapping a regulatory region) or indirect. **b** (top) Circular layout visualization of the reconstructed DNMT3B-dependent transcriptional regulatory network. Dark orange nodes are TFs, green nodes are target genes. (bottom) Node ranking on the basis of their out-degree centrality (i.e. number of regulated genes). Black dots represent the top 1%. DNMT3B direct TF names are depicted in red, indirect in blue. **c** Plot of TFs activity on target genes, measured as their enrichment among DEGs between 3BKO and WT samples using Fisher’s exact test. The *x*-axis reports the −log10(*P*-value). Dot size represents the odds ratio. Dot colour represents statistical significance above (white)/below (black) 0.05. **d** Genome browser view showing the WGBS, ChIP-seq and RNA-seq signal profiles across differentiation (ESC-EpiLC-ME) for WT and 3BKO cells on a ~40 kb window surrounding *Sox2* gene locus. Four DMRs identified as DNMT3B de novo target DMRs are present in this region, named as DMRs [1–4] (depicted in red and indicated in the rectangles). These DMRs overlap with the *Sox2*-associated super enhancer (identified by H3K27ac ChIP-seq signals and previously annotated in ref. ^42^. Annotations for regulatory regions (promoters/typical and super enhancers, as defined by ChIP-seq data), CpG islands and ENCODE candidate *cis-*regulatory elements (ccREs) for mouse mm10^64^ are also reported. **e** Quantification of CpG DNAme (as %) from WGBS of the four identified DMRs [1–4] in *Sox2* locus, in WT and 3BKO cells (average of *n* = 2 biological replicates for each genotype or clone at each time point). Horizontal line indicates median, box indicates the interquartile range (IQR) and whiskers denote the 1.5 × IQR. **f** *Sox2* gene expression levels from RNA-seq (RPKM) over the differentiation time course, in WT and 3BKO cells (***p < 0.001). Bars represent normalized RPKM values, averaged by replicates/conditions (*n* = 2 biological replicates for each genotype or clone at each time point, shown as dots). Error bars represent standard errors. **g** Representative IF of Sox2 in WT and 3BKOs at ME48h stages. Quantification of Sox2+ cells over DAPI is reported as a barplot on the right (***p < 0.001, ANOVA test). Bars indicate mean ± SEM of *n* = 3 independent experiments for each genotype or clone, shown as dots). Error bars represent standard errors.

To identify key TFs involved in the impaired differentiation program of 3BKO cells, we reverse-engineered the DNMT3B-dependent regulatory network of TFs (Fig. 6a). To this end, we leveraged our integrated analysis of the transcriptome, methylome, and histone modifications associated to active or primed regulatory regions together with the known information about TF targets available from TRRUSTv2 and ChEA3^40,41^ databases. The resulting network was composed of 3282 edges and 1358 nodes, with 102 of them being TFs. Network’s node prioritisation by out-degree centrality (i.e. how many genes are regulated by each TF) revealed the presence of *Sox2* as direct target of DNMT3B, among the top 1% central nodes (Fig. 6b). Moreover, local analysis of DNMT3B-direct TF nodes, measuring the enrichment of their target genes among the DEGs in our system, revealed *Sox2* as the top-scoring TF (Fig. 6c).

For these reasons, we decided to focus our attention on *Sox2* as a putative DNMT3B direct target gene, acting upstream of the regulatory hierarchy driving the observed phenotype. Within the genomic region surrounding *Sox2* annotated as super enhancer^42^, we identified one DMR upstream of *Sox2* (DMR1) and 3 DMRs downstream (DMR2–4) that acquired DNAme at the EpiLC stage and were all hypomethylated in the 3BKO compared to WT (Fig. 6d, e). In parallel, the expression of *Sox2* decreased from ESCs to EpiLCs as expected by exiting the ESC pluripotent stage and significantly increased in the 3BKO samples after 48h from the ME induction both at the RNA (Fig. 6d, f) and at the protein level as shown by IF (Fig. 6g).

**Fig. 7:**
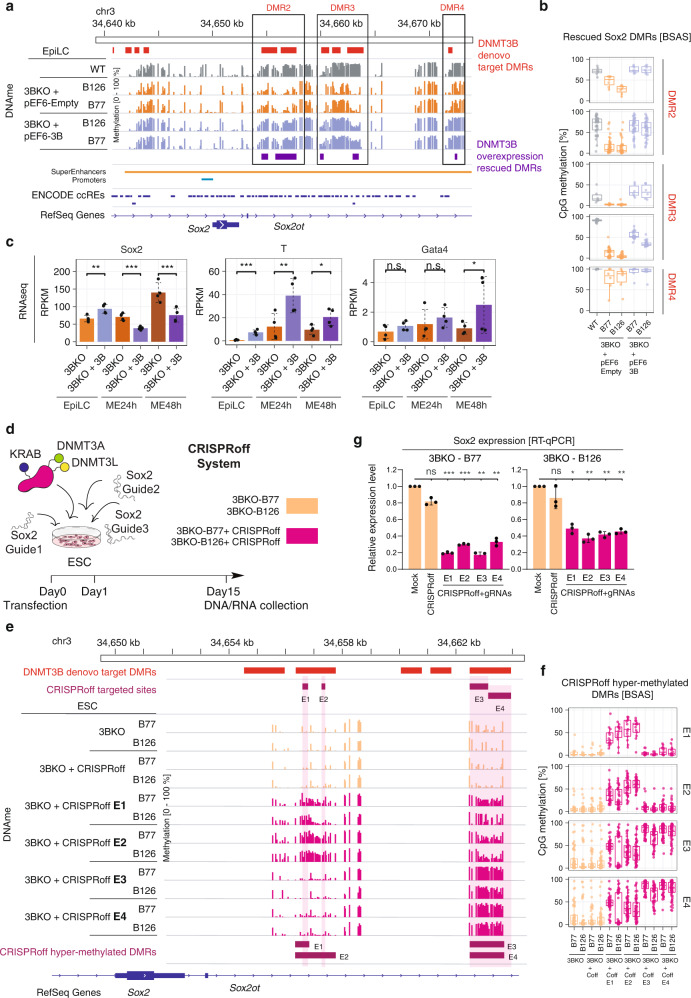
The expression of Sox2 is inhibited by DNAme on *Sox2* super-enhancer. **a** Genome browser view of the BSAS signal profiles after DNMT3B overexpression in 3BKO clones at the EpiLC stage on a ~40 kb window surrounding *Sox2* gene locus. Signal profiles for WT, 3BKO + Empty vector (control) and 3BKO + 3B (Rescued) cells are reported. Three out of four DNMT3B de novo target DMRs (depicted in red), i.e., DMR2,3 and 4, displayed significant rescue of the DNAme levels upon DNMT3B overexpression (depicted in purple). **b**, Boxplots showing quantification of CpG DNAme (as %) from BSAS of the three identified DMRs [2, 3, 4] in *Sox2* locus displaying significant rescue upon DNMT3B overexpression, in 3BKO + Empty vector (control) and 3BKO + 3B cells (Rescued) (*n* = 2 independent clones for each condition). Horizontal line indicates median, box indicates the interquartile range (IQR) and whiskers denote the 1.5 × IQR. **c** Barplots of gene expression leves from RNA-seq of the representative lineage markers *Sox2* (neuro-ectoderm), *T* (mesoderm) and *Gata4* (endoderm) in DNMT3B overexpression and 3BKO control samples across EpiLC-ME differentiation. **d** Schematic of 14-day CRISPRoff protocol indicating the timing and the steps. **e** Genome browser view of the BSAS signal profiles in 3BKO control samples and 3BKO transfected with CRISPRoff and *Sox2* enhancers (E) specific guides on a ~15 kb window surrounding *Sox2* gene locus. An increase in DNAme levels is observed at the targeted sites (E1–E4, depicted in purple). **f** Boxplots showing quantification of CpG DNAme (as %) from BSAS of the CRISPRoff hyper-methylated DMRs in the *Sox2* locus (*n* = 2 independent clones for each condition). Horizontal line indicates median, box indicates the interquartile range (IQR) and whiskers denote the 1.5 × IQR. **g** Bar graphs of normalised *Sox2* expression levels from quantitative RT-qPCR of cells transfected with CRISPRoff and gRNAs targeting the four super enhancer elements (E1–E4). Each dot represents one experimental replicate. (ANOVA test, ***p < 0.001, **p < 0.01, *p < 0.05, ns: not significant).

We sought to analyse whether the Sox2 super-enhancer function was directly determined by DNMT3B-dependent DNAme in the DMRs identified in this study. To this end, we performed locus specific DNAme assay by Bisulfite Amplicon Sequencing (BSAS) (Supplementary Fig. 10a) on DNMT3B-rescued samples at the EpiLC stage, comparing to WT and empty vector samples as control. As evident from the DNAme profiles and their quantification (Fig. 7a, b), the reintroduction of DNMT3B, but not the empty vector, could restore the DNAme to the original WT level in the DMR 2, 3 and 4. The re-methylation induced a decrease in *Sox2* expression and the increase of the expression of the meso-endoderm markers *T* and *Gata4* (Fig. 7c).

To verify whether downregulation of *Sox2* was per se sufficient to recapitulate the phenotype in 3BKO cells we performed loss-of-function experiments by silencing Sox2 using two independent shRNAs in both 3BKO EpiLCs clones and induced them to differentiate into ME (Supplementary Fig. 10b). After 48 h, we observed a significant downregulation of *Sox2* expression with both *Sox2*-targeting constructs with respect to the control. Notably, expression profiling of key meso-endoderm markers by RT-qPCR revealed a significant upregulation of both T and Gata4 in Sox2-silenced 3BKO cells (Supplementary Fig. 10b–d).

To further verify whether the DNAme at these specific regions is sufficient to regulate *Sox2* transcription, we employed the CRISPRoff system to methylate the DNA at specific sites of the super-enhancer (E1–E4) in 3BKO cells (Fig. 7d and Supplementary Fig. 10e) and analysed the effect of DNAme on *Sox2* gene expression by RT-qPCR. We used three guides for each region (Fig. 7e) and assessed the DNAme levels by BSAS, using as negative controls 3BKO cells either untransfected or transfected with the CRISPRoff only. We observed an increase of DNAme levels at the targeted sites upon transfection with the specific guides, as evident from the DNAme profiles and their relative quantitation (Fig. 7e, f). *Sox2* expression was consistently downregulated upon the establishment of DNAme at these regions (Fig. 7g).

Altogether, these results demonstrate the key role played by DNMT3B-dependent downmodulation of *Sox2* for the proper establishment of the meso-endodermal transcriptional program.

## Discussion

DNAme in the early stages of mouse embryo development is established by the de novo DNA methyltransferases at the exit from pluripotency during differentiation into somatic lineages.

Here we report that the de novo DNMTs play distinct roles in the regulation of cell differentiation with DNMT3B playing a major role in cell specification to prime epiblast cells towards ME.

Analysis of the EBs model of unbiased differentiation of EBs revealed that *Dnmt3a* knockout cells can differentiate in both ectoderm and ME, while *Dnmt3b* knockout shows a reduced number of ME compared to Ectodermal cells, suggesting a specific impairment of the ME trajectory.

Our results are in line with the early embryonic lethality observed in vivo in *Dnmt3b* knockout embryos by defects in ME-derived tissues, while *Dnmt3a* knockout mice only die after birth^12,13,43,44^. Nevertheless, the stronger phenotype that we observe in vitro could be ascribed to the lack of compensatory mechanisms that do occur in complex organisms in vivo, but might be absent in the in vitro culture.

These data were confirmed by the induction of EpiLCs toward the ME fate, which demonstrated that the lack of DNMT3B in EpiLCs results in the aberrant upregulation of ectodermal genes affecting the differentiation toward the ME lineage.

In agreement with previous data, we observed that the increase of DNAme in EpiLCs is mainly due to DNMT3B upregulation and is more pronounced than in vivo epiblasts^35,37^. During the in vitro differentiation, the majority of DMRs showed a consistent dynamic which is consistent with the in vivo process, despite a small subset of regions that display in vitro-specific hypermethylation (Supplementary Figs. 4 and 6).

Interestingly, the lack of DNMT3B does not prevent the establishment of EpiLCs, rather inhibits their further differentiation into the ME lineage, in agreement with the idea that DNAme, deposited at the epiblast stage, is responsible for cell priming to ensure the correct cell specification at later stages.

The data presented in this work demonstrate that DNMT3B plays a key role in DNAme-dependent priming of EpiLCs toward ME. By WGBS we found that 3BKO cells fail to methylate the DNA at enhancers connected to ectodermal development genes, which are not switched off in the following days resulting in their increased neuro-ectodermal differentiation.

The hypomethylated loci in 3BKO are largely associated with markers of neuro-ectoderm differentiation such as *Sox1, Sox2, Olig3,* and *Tubb3*, in agreement with data obtained from early stages of mouse differentiation^10^. This suggests that, in order to differentiate into meso-endoderm, EpiLCs should repress the chromatin of a number of ectoderm enhancers that at this stage are open and demethylated and should be decommissioned in the cells primed to be able to differentiate into meso-endoderm^42,45^. Thus, DNMT3B-dependent DNAme establishes the meso-endodermal epigenetic landscape by repressing the expression of key TFs that would otherwise induce the default differentiation into neuro-ectoderm.

Our analysis of DNMT3B direct targets focused on *Sox2*, a well-known master regulator that acts antagonistically to T to promote neural differentiation^46^. Accordingly, we found that Sox2 silencing in 3BKO EpiLCs, restores the expression of the meso-endoderm markers during their further differentiation. By analysis of DNAme, we observed that *Sox2* super-enhancer shows a significant reduction of methylation in 3BKO EpiLCs, which results in *Sox2* upregulation. Importantly, *Sox2* expression can be inhibited by rescuing the expression of DNMT3B in 3BKO cells thus confirming that DNMT3B-dependent DNAme is required for meso-endodermal differentiation. Furthermore, by site-specific CRISPR-based DNAme targeting we identified the *Sox2* enhancer elements whose DNA methylation is responsible for its downregulation.

Our data provide functional characterization of the de novo DNMT3B during lineage determination establishing that DNMT3B-dependent methylation is essential to prime EpiLCs for their further differentiation into the ME lineages.

## Methods

### Cell culture

E14 mouse WT and mutant (*Dnmt3a*^−/−^ and *Dnmt3b*^−/−^)^18,29^ ESCs were cultured in high-glucose DMEM (Euroclone_#ECM0728L) supplemented with 18% FBS (Sigma_#ES-009-B), 0.1 mM MEM non-essential amino acids (Invitrogen_#11140050), 1 mM sodium pyruvate (Invitrogen_#11360070), 0.1 mM β-mercaptoethanol (Sigma_#M3148), 1500 U/ml Leukemia Inhibitory Factor (LIF; Millipore_#LIF2010), 25 U/ml penicillin, and 25 μg/ml streptomycin (Invitrogen_#15070063). All cell lines were mycoplasma-negative (Mycoalert, Lonza_#LT07-318).

### Embryoid body formation

To induce formation of EBs, WT and mutant ESCs were gently dissociated using trypsin/EDTA (Invitrogen_#R001100) to form a single-cell suspension. 50 μl of cell suspension containing a total of 100 cells were pipetted into each well of the ultra-low attachment 96-well plates (Corning_#7007). 150 μl of EB formation medium were then added to each well. EB formation medium is composed by Alpha-MEM (LONZA_#BE02-002F) medium supplemented with 10% KOSR (GIBCO_#10828-028), 5% FBS (Sigma_#ES-009-B), 1% MEM non-essential amino acids (Invitrogen_#11140050), 1 mM sodium pyruvate (Invitrogen_#11360070), 1% glutamine (Invitrogen_#A2916801), 0.1 mM β-mercaptoethanol (Sigma_#M3148), 25 U/ml penicillin and 25 μg/ml streptomycin (Invitrogen_#15070063). Medium was changed every 3 days until the end of the differentiation. Many EBs from different wells were pooled and collected for single cell sequencing and immunostaining at differentiation time points.

### Histology and immunostaining on sections

Embryoid bodies at day 9 were collected and embedded in OCT compound (Bio-Optica). Frozen tissue blocks were sectioned (10 μm) with a CM3050S Leica cryostat (Leica Mycrosystems) and kept at −80 °C. For immunostaining, slides were thawed and fixed in 4% paraformaldehyde (PFA, Sigma-Aldrich) for 10 min at room temperature (RT). Samples were washed three times in PBS for 5 min and incubated in Gelatin Blocking (GB) solution (2% fish gelatin (Sigma Aldrich), 5% foetal bovin serum (Thermo Fisher), 1% BSA (Sigma Aldrich), 0.3% Triton X-100 (Sigma Aldrich) in PBS) for 1 h at RT. Sections were again washed three times with PBS. The following primary antibodies were resuspended in GB solution and incubated overnight at 4 °C: anti-Sox2 (clone 245610) (Mouse IgG2a, 1:200, BD Biosciences_#560291); anti-Brachyury (Goat IgG, 1:200, Biotechne_#AF2085); anti-TUJ1 (Rabbit IgG, 1:500, BioLegend_#MRB_435P). After 15 min of PBS washes, samples were incubated with secondary antibodies 1 h at RT. Alexa Fluor-568- or 488- conjugated secondary antibodies (Life Technologies) were used at 1:1000 dilution. 6-diamidine-20-phenylindole dihydrochloride (DAPI) (Thermo-Fisher, diluted 1 μg/mL) was used for nuclear staining and incubated in the dark for 5 min at RT. Slides were mounted with ProLong Glass Antifade Mountant (Invitrogen).

### EpiLCs induction from ESCs

EpiLCs induction was modified from^47^. Briefly, ES cells were gently dissociated using trypsin/EDTA, and a single-cell suspension was plated onto Geltrex (GIBCO_#A1413202)-coated plates at a density of 5000 cells/cm^2^ in ESC growth medium. One day after plating, EpiLC induction started by adding the N2B27 medium supplemented with 20 ng/ml ActivinA (GIBCO_#PHC9564) and 12 ng/ml bFGF (GIBCO_#PHG0026). The cells were splitted 1:3 in small clumps using 1 mg/ml Collagenase IV (GIBCO_#17104019). Medium was changed daily. EpiLCs were collected for DNA, RNA and protein analyses after 14 days of induction.

N2B27 medium is composed by 50% advanced DMEM/F12 (GIBCO_#12634028) and 50% Neurobasal medium (GIBCO_#21103049), supplemented with 0.5% N2 Supplement (GIBCO_#17502048), 1% B27 Supplement (GIBCO_#17504044), 0.033% BSA solution (SIGMA_#A9647), 50 µM β-mercaptoethanol (Sigma_#M3148), 2 mM Glutamax (GIBCO_#35050038), 25 U/ml penicillin and 25 μg/ml streptomycin (Invitrogen_#15070063).

### EpiLCs differentiation towards meso-endoderm lineage

Meso-endoderm directed lineage specific differentiation was carried out as previously described^34^. Briefly, EpiLCs were plated as small clumps onto Geltrex-coated plates in EpiLCs medium for 24 h. The day after, medium was replaced with N2B27 medium consisted of 50% advanced DMEM/F12 (GIBCO_#12634028) and 50% Neurobasal medium (GIBCO_#21103049), supplemented with 0.5% N2 Supplement (GIBCO_#17502048), 1% B27 supplement minus Vitamin A (GIBCO_#12587010) and 3 μM iGSK3β (SIGMA_#CHIR99021). Cells were fed daily until the end of differentiation. Cells were collected for DNA, RNA and protein analyses at 24 and 48 h of the differentiation.

### Rescue of the meso-endoderm phenotype in *Dnmt3b* mutant cells

*Dnmt3b* full-length construct was obtained by PCR and tagged N-terminally with Avitag. The expression construct was cloned into pEF6/V5-His vector (Invitrogen_#K961020). *Dnmt3b*^−/−^ stem cells were transfected with a plasmid expressing *Dnmt3b* using Lipofectamine 2000 Transfection Reagent (Invitrogen_#11668500) and cultured for 10 days in presence of blasticidin. Resistant cells were expanded for EpiLC induction and meso-endoderm differentiation. Dnmt3b expression was confirmed by western blotting with anti-Dnmt3b of the total lysates. Cells were collected for DNA and RNA analyses at EpiLC state, 24 and 48 h of the meso-endoderm differentiation.

### *Sox2* silencing in *Dnmt3b* mutant cells

Custom shRNAs against *Sox2* were constructed using the TRC hairpin design tool (http://www.broadinstitute.org/rnai/public/seq/search), and designed to target the following sequences:

5′-ACCAATCCCATCCAAATTAAC-3′ (shRNA1)

5′-GCACAGTTTGAGATAAATAAA-3′ (shRNA2)

Hairpins were cloned into pLKO.1 vector (Addgene plasmid_#10878) and each construct was verified by sequencing. Oligonucleotide sequences for shRNA cloning are reported in Supplementary Data 8. For *Sox2* silencing, EpiLCs were plated in 6-well plates and the day after they were transfected with 5 μg of the specific shRNA construct using Lipofectamine 2000 Transfection Reagent (Invitrogen_#11668500) in accordance with the manufacturer’s protocol, and maintained in meso-endoderm differentiation medium for 48 h. Cells were collected for RNA and protein analyses at 48 h of the meso-endoderm differentiation.

### CRISPRoff targeting on *Sox2* super enhancer

Transient transfection experiments in ES cells were performed in six-well plates using Lipofectamine 2000 Transfection Reagent (Invitrogen_#11668500). Cells at 70–80% confluency were transfected with 2.5 μg of plasmid encoding CRISPRoff^48^ (Addgene plasmid_#167981) and 800 ng of plasmid encoding each sgRNA. Cells were monitored for BFP (CRISPRoff) expression 24 h after transfection. Cells were passed every 3 days following daily medium changes. The cells were collected for DNA and RNA analyses after 14 days. The sgRNAs targeting *Sox2* enhancers (E1, E2, E3, E4) were listed on Supplementary Data 8.

### Protein extraction and Western blotting

For total cell extracts, cells were resuspended in F-buffer (10 mM TRIS-HCl pH 7.0, 50 mM NaCl, 30 mM Na-pyrophosphate, 50 mM NaF, 1% Triton X-100, anti-proteases) and sonicated for 3 pulses. Extracts were quantified using bicinchoninic acid assay (Pierce™ BCA Protein Assay Kit; Thermo Scientific_#23227) and were run on SDS-polyacrylamide gels at different percentages, transferred to nitrocellulose membranes and incubated with specific primary antibodies overnight. Western blotting was acquired with Bio-Rad ChemiDoc imaging system. The primary antibodies used for western blot were listed in Supplementary Data 8. Uncropped western blot gels are provided in Supplementary Fig. 11.

### Immunofluorescence

Immunofluorescence analysis was performed on Geltrex-coated eight-well chambered glass coverslip. Cells were fixed with 4% paraformaldehyde for 15 min at room temperature. Permeabilization was performed in 0.3% Triton X-100 in PBS for 5 min at room temperature, and then the cells were blocked in 1% BSA in PBS at room temperature for 1 h. Cells were stained with primary antibodies for 1 h at room temperature. The primary antibodies used for immunofluorescence were listed in Supplementary Data 8. Secondary antibodies (Alexa, Invitrogen) were applied for 1 hat room temperature. Nuclei were stained with DAPI (Invitrogen_#D21490). Images were acquired using a Leica TCS SP5 Confocal microscope and LAS AF Lite software.

### Single cell RNA-seq library preparation

For single cell library prep, 50 EBs for each clone of each genotype at each time point were collected and dissociated using trypsin/EDTA for 5 min at room temperature. Cells were then washed with PBS, and the resulting cell suspension was used to sort individual live cell in 96 well plate. Full length single cell RNA-seq was performed using a modified version of the Smart-seq2 protocol^49^ as in ref. ^31^. Briefly, individual cells were sorted into 96 well plates containing lysis buffer in presence of RNase inhibitor, dNTPs and oligodT. Reverse transcription of the polyadenylated RNA was performed with SuperScriptII and Template Switching Oligos. The resulting cDNA was amplified with 25 cycles of PCR and libraries were prepared for sequencing with miniaturized NexteraXT Illumina protocol. Libraries were sequenced on Illumina NextSeq 500 System (single-end 75 bp reads), reaching a median of ~ 578,000 generated reads per cell.

### RNA extraction for RNA-seq library preparation and RT-qPCR analysis

Total RNA was extracted by using QIAzol Lysis Reagent (Qiagen_#79306), according to the manufacturer’s protocol. For library preparation, the quantity and quality of the starting RNA were checked by Qubit and Bioanalyzer (Agilent). 1 μg of total RNA was subjected to poly(A) selection, and libraries were prepared using the TruSeq RNA Sample Prep Kit (Illumina) following the manufacturer’s instructions. Libraries were sequenced on Illumina NextSeq 500 System (single-end 75 bp reads).

Real-time PCR was performed using the SuperScript III Platinum One-Step RT-qPCR Kit (Invitrogen_#11732088) following the manufacturer’s instructions. Three technical replicates were carried out for all RT-qPCR analysis. The gene expression levels were normalised to β-actin gene. Oligonucleotide sequences are reported in Supplementary Data 8. RT-qPCR data were acquired with Rotor-Gene Q series software.

### Chromatin Immunoprecipitation (ChIP) sequencing

ChIP experiments were performed as previously described^50^. Approximately 2 × 10^7^ cells were cross-linked by 1% formaldehyde for 10 min at RT, quenched with 0.125 M glycine for 5 min and then washed twice in cold PBS. The cells were resuspended in Lysis Buffer (50 mM Hepes-KOH pH 7.5, 140 mM NaCl, 1 mM EDTA, 10% Glycerol, 0.5% NP-40, 0.25% Triton X-100 and protease inhibitor) to disrupt the cell membrane and in Nuclei Lysis Buffer (10 mM Tris-HCl pH8.0, 200 mM NaCl, 1 mM EDTA, 0.5 mM EGTA and protease inhibitor) to isolate nuclei. Nuclei were then resuspended in SDS ChIP Buffer (20 mM Tris-HCl pH 8.0, 10 mM EDTA, 1% SDS and protease inhibitors). Extracts were sonicated using the BioruptorH Twin (Diagenode) for two runs of 10 cycles [30 s “ON”, 30 s “OFF”] at high power setting. Cell lysate was centrifuged at 12,000 × *g* for 10 min at 4 °C. The supernatant was diluted with ChIP Dilution Buffer (20 mM Tris-HCl pH 8.0, 150 mM NaCl, 2 mM EDTA, 1% Triton) before immunoprecipitation step. The beads (Dynabeads™ Protein G; Invitrogen_#10003D) were saturated with 1% BSA/PBS and the samples were incubated with 2 μg of antibody (See Supplementary Data 8) overnight at 4 °C on a rotator. Next day samples were incubated with saturated beads for two h at 4 °C on a rotator. Successively, immunoprecipitated complexes were washed five times with RIPA buffer (50 mM Hepes-KOH pH7.6, 500 mM LiCl, 1 mM EDTA, 1% NP-40, 0,7% Na-Deoxycholate) at 4 °C for 5 min each on a rotator. Elution Buffer was added and incubated at 65 °C for 15 min. The decrosslinking was performed at 65 °C overnight. De-crosslinked DNA was purified using QIAQuick PCR Purification Kit (Qiagen_#28106) according to the manufacturer’s instructions.

10 ng of ChIP eluted sample were used to prepare the library following the manufacturer’s instructions of NEBNext® ChIP-Seq Library Prep Reagent Set for Illumina® (NEB_#E6240L). Libraries were sequenced on Illumina NextSeq 500 System (single-end 75 bp reads).

### Bisulfite amplicon sequencing (BSAS)

Genomic DNA was extracted from cells using the DNeasy Blood and Tissue kit (Qiagen_#69506) following the manufacturer’s instructions. For each condition, 1 μg genomic DNA was subjected to bisulfite conversion and cleanup according to the manufacturer’s instructions using the EpiTect Bisulfite kit (Qiagen_#59104), eluting to 40 μl. 3 μl eluate was used for individual amplicons PCR amplification using AccuPrime™ Taq DNA Polymerase System (Invitrogen_#12346086). Amplicons were then purified using a MinElute PCR Purification Kit (Qiagen_#28006) and eluted to 15 μl. The *Sox2* super enhancer amplicons were amplified using the primer pairs listed in Supplementary Data 8. Amplicons were sequenced on the Illumina NextSeq 500 Platform.

### Whole genome bisulfite-seq (WGBS) library preparation and sequencing

For WGBS library preparation, 5 μg of genomic DNA were sheared using a Bioruptor Pico sonicator (Diagenode) for two runs of twenty cycles [30 s ‘’ON”, 30 s ‘’OFF”] at high power setting to obtain ~200 bp fragments. Sonicated DNA was then end-repaired, dA-tailed, and ligated to methylated adapters, using the Illumina TruSeq DNA Sample Prep Kit, following the manufacturer’s instructions. Adapter-ligated DNA was loaded on an E-Gel Size select 2% agarose pre-cast gel (Invitrogen_#G661012), and a fraction corresponding to fragments ranging from 250 bp to 350 bp was recovered. Purified DNA was then subjected to bisulfite conversion using the EpiTect Bisulfite Kit (Qiagen_#59104) following the manufacturer’s instructions. Bisulfite-converted DNA was finally enriched by 15 cycles of PCR using PfuTurbo Cx HotStart DNA Polymerase (Agilent_#600410). Libraries were sequenced on Illumina Novaseq 6000 System, generating ~500  × 10^6^ 100 bp paired-end reads and an average coverage depth of 30X per base in each sample.

### Data analysis

#### Single cell RNA-seq data analysis

Following quality controls (performed with FastQC v0.11.2 (https://www.bioinformatics.babraham.ac.uk/projects/fastqc), sequencing reads were processed with Trim Galore! v0.5.0 (https://www.bioinformatics.babraham.ac.uk/projects/trim_galore) to perform quality and adapter trimming (parameters:*–stringency 3 –q 20*). Trimmed reads were next aligned to the mouse reference genome (mm10/GRCm38 Ensembl release 84) using STAR v2.7.1a^51^ with options:*–outFilterMultimapNmax 10–outFilterMultimapScoreRange 1 –outFilterMismatchNmax 999 –outFilterMismatchNoverLmax 0.04*. Gene expression levels were quantified with featureCounts v1.6.1 (https://subread.sourceforge.net/, options: *-t exon -g gene_name*) using the GENCODE Release M23 annotation. Multi-mapped reads were excluded from quantification.

The following criteria were applied to exclude low-quality cells from subsequent analyses: <50,000 assigned reads; <2000 detected genes; more than 50% of reads assigned to mitochondrial genes, resulting in 1451 high quality cells, with a median of >250,000 reads per cell assigned to annotated transcripts. Gene expression counts were next analyzed using the Seurat v4.0.1 package^32^. Read counts were first coverage-normalized and log-transformed (*NormalizeData* function with default parameters); next, variance modelling for feature selection was carried out using the *FindVariableFeatures* function (parameters: *selection.method* = *”vst”, nfeatures* = *2000*), selecting the top 2000 variable genes for the subsequent analyses. The selected features were scaled and centred using the *ScaleData* function, which also allowed to regress-out unwanted sources of variability (i.e. library preparation batch, percentage of mitochondrial reads, by setting the parameter *vars.to.regress*). For the second experiment (Supplementary Fig. 1), batch effects were removed following the Seurat integration workflow (implemented via the *SelectIntegrationFeatures*, *FindIntegrationAnchors* and *IntegrateData* functions, with parameters: *k.score* = *40, k.weight* = *20*). Principal component analysis (PCA) was then performed using the *runPCA* function (default parameters), retaining 8 meaningful components—as determined by inspection of the elbow point in the standard deviation graph (*ElbowPlot* function)—for cell clustering, which was next performed with the Louvain method implemented in the *FindNeighbors* and *FindClusters* functions (parameters: *resolution* = *0.8*). UMAP embedding was performed using the *runUMAP* function (parameters: *reduction* = *“pca”*). Cluster marker genes were then identified using the logistic regression approach implemented in the *FindAllMarkers* function (parameters: *test.use* = *“LR”, only.pos* = *TRUE, min.pct* = *0.05, logfc.threshold* = *0.1*). Gene set over-representation analysis was performed for the top markers list of each cluster with the gProfileR package (*gprofiler* function, parameters: *max_set_size* = *750*). Single cell trajectory analysis was performed on top of the Seurat pipeline using Monocle3^33^. To this end, the Seurat-processed object was converted to a Monocle3 object using the *as.cell_data_set* function (implemented in the SeuratWrappers package). Partitions were calculated using the *cluster_cells* function (parameter *reduction_method* = *“UMAP”*), while clusters were retained from the Seurat analysis. Pseudotime analysis was then carried out by reverse graph embedding using the *learn_graph* and *order_cells* functions, setting as the starting point of the trajectory the earliest principal point of the epiblast-like cells cluster. Two differentiation paths (i.e. Epi-to-Ect, Epi-to-ME) were then retrieved by grouping the relevant cell clusters along the principal graph. DEGs along the two branches were identified using the graph-autocorrelation analysis implemented in the *graph_test* function (parameters: *neighbor_graph* = *“principal_graph”*). The smoothed relative expression in each branch for the significantly pseudotime-dependent genes (FDR < 0.01) was hierarchically clustered and visualised as heatmap using ComplexHeatmap^52^. Gene set over-representation analysis was performed with the clusterProfiler package.

### RNA-seq data analysis

Following quality controls (performed with FastQC v0.11.2), sequencing reads were aligned to the mouse reference genome (mm10/GRCm38 Ensembl release 84) using HiSat2 v2.2.1^53^. Pre-built indexes based on the Ensembl transcript annotation (release 84) for guided alignment to transcriptome were retrieved from the HiSat2 web site (https://cloud.biohpc.swmed.edu/index.php/s/grcm38_tran/download). Gene expression levels were quantified with featureCounts v1.6.1 using the Ensembl release 84 transcript annotation (ftp://ftp.ensembl.org/pub/release-84/gtf/mus_musculus/Mus_musculus.GRCm38.84.gtf.gz). Multi-mapped reads were excluded from quantification.

Gene expression counts were next analysed using edgeR v3.32.1^54^. For the WT/3BKO RNA-seq experiment (i.e. Fig. 2), lowly expressed/not detected genes (i.e. 1 RPKM in <2 samples) were filtered out, obtaining a total of 16,755 expressed genes for downstream analysis. Normalization factors were calculated using the trimmed mean of M-values (TMM) method (*calcNormFactors* function) and RPKM were computed using normalized library sizes and gene lengths from the Ensembl release 84 annotation (*rpkm* function). Principal Component Analysis (PCA) was performed using the *prcomp* R function (parameters: *scale* = *TRUE, center* = *TRUE*), using the top 2500 variable genes. Following dispersion estimation (*estimateDisp* function, *robust* = *TRUE*), an ANOVA-like test was implemented by fitting a Generalized Linear Model (GLM) to all sample groups (*glmFit* function) and performing Quasi-Likelihood *F*-test (*glmQLFTest* function) in order to identify the genes that were significantly varying during the differentiation time course (i.e. DEGs in any of the sample groups during the time course, using the ESC-WT condition as baseline in the design matrix formula). The resulting 4624 genes (|logFC| >=  1.5 and FDR < = 0.001) were used for clustering of gene expression profiles with K-means (*kmeans* R function, parameters: *centers* = *4, iter.max* = *25, nstart* = *100*) followed by hierarchical clustering (parameters: *method* = *”single”, distance* = *“euclidean”*). RPKM values were scaled as Z-scores across samples before computing distances. The optimal number of K-means clusters (*n* = 4) was estimated using the within-cluster sum of squares methodology. Gene expression heatmaps were generated using the ComplexHeatmap R package^52^. Gene set over-representation analysis was performed for each cluster with the gProfileR (https://cran.r-project.org/web/packages/gProfileR/index.html) and ClusterProfiler (https://bioconductor.org/packages/release/bioc/html/clusterProfiler.html) packages, using all the expressed genes as background. DEGs between WT and 3BKO cells at each time point were obtained from the same GLM, comparing each contrast with the Quasi-Likelihood *F*-test (|logFC| >= 1 and FDR < = 0.05). For the DNMT3B ectopic expression experiment (i.e. Fig. 3), the same processing pipeline was applied, and DEGs were identified by comparing 3BKO + pEF6-3B and 3BKO + pEF6-Empty samples at each time point (|logFC |>= 0.5 and FDR < = 0.05). For the in vitro/in vivo comparisons, dataset integration was performed using ComBat^55^.

### ChIP-seq data analysis

Publicly available (ENCODE) and newly generated ChIP-seq data for the histone marks H3K4me3, H3K4me1, and H3K27ac were used to annotate the set of putative regulatory elements arising during differentiation. Following quality controls (performed with FastQC v0.11.2), sequencing reads were aligned to the mouse reference genome (mm10/GRCm38) using Bowtie v2.3.4.1^56^ (options: -q –local). Duplicated alignments (identified by Picard MarkDuplicates, https://broadinstitute.github.io/picard) and low-quality alignments/multi-mapping reads were excluded using SAMtools^57^. Coverage tracks were generated from filtered alignments using the deepTools suite^58^. Immunoprecipitation and corresponding control (Input DNA) datasets were treated identically. Peak calling was performed using MACS v2.1.1^59^. The read extension size (ES) was estimated by cross-correlation using the *phantompeakqualtools* package. Input-normalized ChIP-seq signals were obtained using the deepTools^58^. These processing steps were applied to all sample groups. Identification of typical and super enhancer regions was performed with ROSE^60^. Common and time-point specific differentiation enhancers were obtained using the *mergePeaks* utility from the HOMER suite (http://homer.ucsd.edu/homer). Signal profiles over peaks/genomic regions were obtained using the deepTools suite^58^.

### WGBS and BSAS data analysis

Following quality controls, sequencing reads were processed with Trim Galore! v0.5.0 (https://www.bioinformatics.babraham.ac.uk/projects/trim_galore) to perform quality and adapter trimming (parameters:*–stringency 3 –q 20–paired*). Trimmed reads were next aligned to the mouse reference genome (UCSC mm10/GRCm38) using Bismark v0.22.3^61^. The bisulfite-converted genome was created using the *bismark_genome_preparation* utility (parameters:*–genomic_composition–bowtie2*). Read mapping was performed with the *bismark* command (parameters:*–nucleotide_coverage*). Duplicated alignments were removed with the *deduplicate_bismark* utility and methylation calling was carried out using the *bismark_methylation_extractor* utility (parameters:*–ignore 1–bedGraph–counts–gzip*). Genome-wide cytosine methylation reports with the top and bottom strand methylation evidence pooled into a single CpG dinucleotide entity were obtained using the *coverage2cytosine*utility (parameters:–zero_based–gzip–merge_CpG).

DMRs were identified using the DSS R package^62^, performing all pairwise comparisons between differentiation time points of WT samples, and between WT and 3BKO at matching time points. For each comparison, the *DMLtest* function was first run (parameters: *equal.disp* = *FALSE, smoothing* = *TRUE, smoothing.span* = *500*); next, differentially methylated loci were identified with the *callDML* function (parameters: *delta* = *0.1, p.threshold* = *0.001*); finally, DMRs were called using the *callDMR* function (parameters: *delta* = *0.2, p.threshold* = *0.05, minCG* = *5, dis.merge* = *100*). The resulting list of DMRs for each relevant set of comparisons (i.e. WT time course and 3BKO versus WT) was combined into one DMRs set, collapsing overlapping regions into a single DMR. For further analysis, only CpG sites with coverage > = 5x and DMRs with *coverage* > *=10*x in all samples were retained, and average DNAme levels for each DMR was calculated using the methylKit package (*regionCounts* and *percMethylation* functions). PCA was performed using the *PCASamples* function of methylKit (parameters: *filterByQuantile* = *T, sd.threshold* = *0.5, sd.filter* = *T*). Samples hierarchical clustering and correlation analysis was performed in 400 bp tiles using the *cor*, *dist* and *hclust* functions in R (parameters: *method* = *“ward.D”*). DMRs clustering was performed using K-means, scaling the DNAme scores before computing distances (*scale* and *kmeans* R functions). DMRs heatmaps were generated using ComplexHeatmap^52^. DMRs annotation to genomic features and genes was performed using BEDtools, GAT^39^ (number of samplings = 1000) and rGREAT^63^ (parameters: *rule* = *“basalPlusExt”, adv_span* = *100*). Gene set over-representation analysis was performed using rGREAT and clusterProfiler packages. For BSAS, the same data processing pipeline was applied, but duplicated reads were not removed, and CpG sites with coverage > *= 10*x were retained in downstream analyses.

### Comparative methylomic analysis

For comparative analysis with the in vivo E6.5 epiblast^37^, PCA and hierarchical clustering was performed using DNAme scores measured in 400 bp tiles. The average DNAme levels on the in vitro DMRs identified between 3BKO and WT EpiLCs was then computed for all samples to determine the set of in vitro DMRs that were consistently hypomethylated in vivo. For comparative analysis with human HUES64, the same procedure for the identification and annotation of DMRs (described above) was applied to human data. The mouse/human gene orthologs map was retrieved from Ensembl BioMart (https://www.ensembl.org/), and the conserved genes were defined as having at least one associated hypomethylated DMR between 3BKO and WT in both species. Annotations of cCREs for mouse (mm10) and human (hg38) were retrieved from ENCODE SCREEN database^64^. Enrichment analysis was performed using clusterProfiler.

### Integrated analysis

Multiple Factor Analysis (MFA) of the RNA-seq and WGBS profiles across differentiation was performed using the FactoMineR package. The DNMT3B-target genes were defined by associating the DNMT3B-DMRs (within 100 kb from the TSS and overlapping a putative regulatory region) to upregulated genes in 3BKO versus WT cells across differentiation. Grouping of early/mid/late genes was performed based on the time point in which the biggest difference in expression occurs between 3BKO and WT cells. Enrichment analysis was performed with clusterProfiler.

### DNMT3B-dependent regulatory network reconstruction

To build the DNMT3B-dependent gene regulatory network, the TF-target regulatory evidence from TRRUSTv2^40^ and ChEA3^41^ databases were integrated. For ChEA3, the intersection between the co-expression-based (ARCHS4_Coexpression.gmt and GTEx_Coexpression.gmt) and ChIP-seq-based (ENCODE_ChIP-seq.gmt, Literature_ChIP-seq.gmt and ReMap_ChIP-seq.gmt) TF-target connections was retained. The resulting network ‘backbone’ was filtered for DEGs across differentiation, and the DNMT3B-direct regulatory evidence was used to classify nodes as direct (i.e. upregulation in 3BKO, downregulation in DNMT3B overexpression, association to at least one 3BKO-hypomethylated DMR overlapping a regulatory region) or indirect. Network metrics calculation and visualisation was performed with the igraph (https://igraph.org/) package (*degree* and *plot.igraph* functions). TF target enrichment among DEGs was performed using the fisher.test R function (parameters: *alternative* = *“g”*).

### Reporting summary

Further information on research design is available in the Nature Portfolio Reporting Summary linked to this article.

## Supplementary information

Supplementary Information

Description of Additional Supplementary Files

Supplementary Data 1

Supplementary Data 2

Supplementary Dataset 3

Supplementary Data 4

Supplementary Data 5

Supplementary Data 6

Supplementary Data 7

Supplementary Data 8

Reporting Summary

Source_Data

## Data Availability

ChIP-seq and DNase-seq data for ESCs were obtained from ENCODE (https://www.encodeproject.org/). The datasets for comparative analyses were retrieved from the Gene Expression Omnibus (GEO) database, with accession codes: GSE63281, GSE76505, GSE137337. The datasets generated in this study are available as raw data in the GEO database with accession code GSE168415. Additional data are provided as Supplementary Tables and Source Data files.

## Code availability

The code used for generating the results is available at https://github.com/andrealauria104/Lauria_et_al_NatComm2022.
