# Supplementary material for: DNMT3B supports meso-endoderm differentiation from mouse embryonic stem cells
Source: Nat Commun. 2023 Jan 23;14:367. doi: 10.1038/s41467-023-35938-x (PMC9871038; doi:10.1038/s41467-023-35938-x)
Supplement: Supplementary file 1 — Supplementary Information [file 41467_2023_35938_MOESM1_ESM.pdf]

Lauria et al.

DNMT3B supports meso-endoderm differentiation from mouse embryonic stem cells.

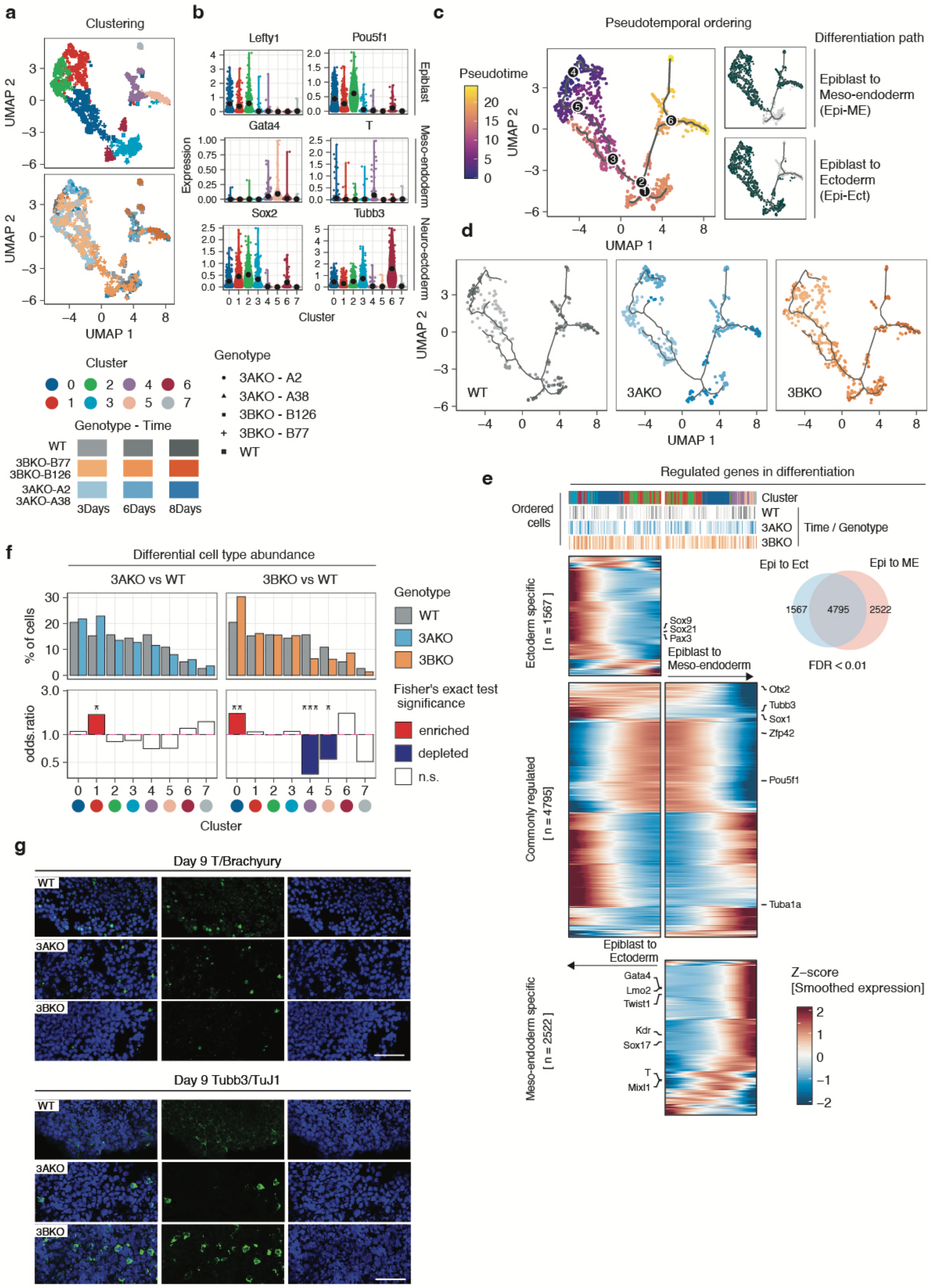

Supplementary Fig. 1

a, UMAP embedding of 965 WT, 3AKO and 3BKO single cell transcriptomes from independent EBs differentiation experiment. Unsupervised clustering, performed using the Louvain algorithm from the Seurat pipeline, identified eight cell clusters, annotated according to the expression of embryonic cell population markers (Supplementary Data 1). Cells are colored by cluster (top panel) and genotype / time of cells' collection (3 Days, 6 Days and 8 Days, bottom panel). 3AKO = shades of blue, 3BKO = shades of orange, WT = shades of grey.

b, Gene expression levels distribution of representative epiblast (*Lefty1*, *Pou5f1*), meso-endoderm (*Gata4*, *T*) and ectoderm markers (*Sox2*, *Tubb3*) in the eight identified cell clusters.

c, Pseudotime analysis of single cell differentiation trajectories with reverse graph embedding[1]. The line plot on the UMAP represents the embedded trajectory graph. Cells are colored according to pseudotime (left panel) and differentiation paths (i.e. Epi-ME, Epi-Ect, right panels).

d, UMAP visualisation of the reconstructed differentiation trajectories for each source cell type (i.e. WT, 3AKO and 3BKO genotype).

e, Heatmap showing the expression patterns of genes differentially regulated in pseudotime along the two differentiation branches, performed via the graph-autocorrelation analysis method [1]. Genes are grouped according to the branch in which they show significant variation (FDR < 0.01, Supplementary Data 2): Epi-to-Ect specific (top cluster), Epi-to-ME (bottom cluster) specific or regulated in both branches (mid cluster). Key marker genes regulated in each group are indicated.

f, Barplots showing the differential cell type abundance in terms of the genotype of origin (i.e. WT, 3AKO and 3BKO) in each of the five identified cell clusters. For each cluster, the relative proportion of mutant cells (3AKO, 3BKO) was compared with WT cells using Fisher's exact test. Top panels report the percentage of cells in each cluster for the indicated comparisons (i.e. 3AKO vs WT, 3BKO vs WT). Bottom panels report the odds ratio from Fisher's exact test, colored for their significance (enriched = red, depleted = blue, non-significant = white) (***p < 0.001, **p < 0.01, *p < 0.05, one-sided).

g, Representative IF for the meso-endodermal marker T/Brachyury and the neuro-ectodermal marker Tubb3/Tuj1 in WT, 3AKO and 3BKO EBs on Day 9 of differentiation. Representative images of three independent experiments are shown. Scale bars 50 μm.

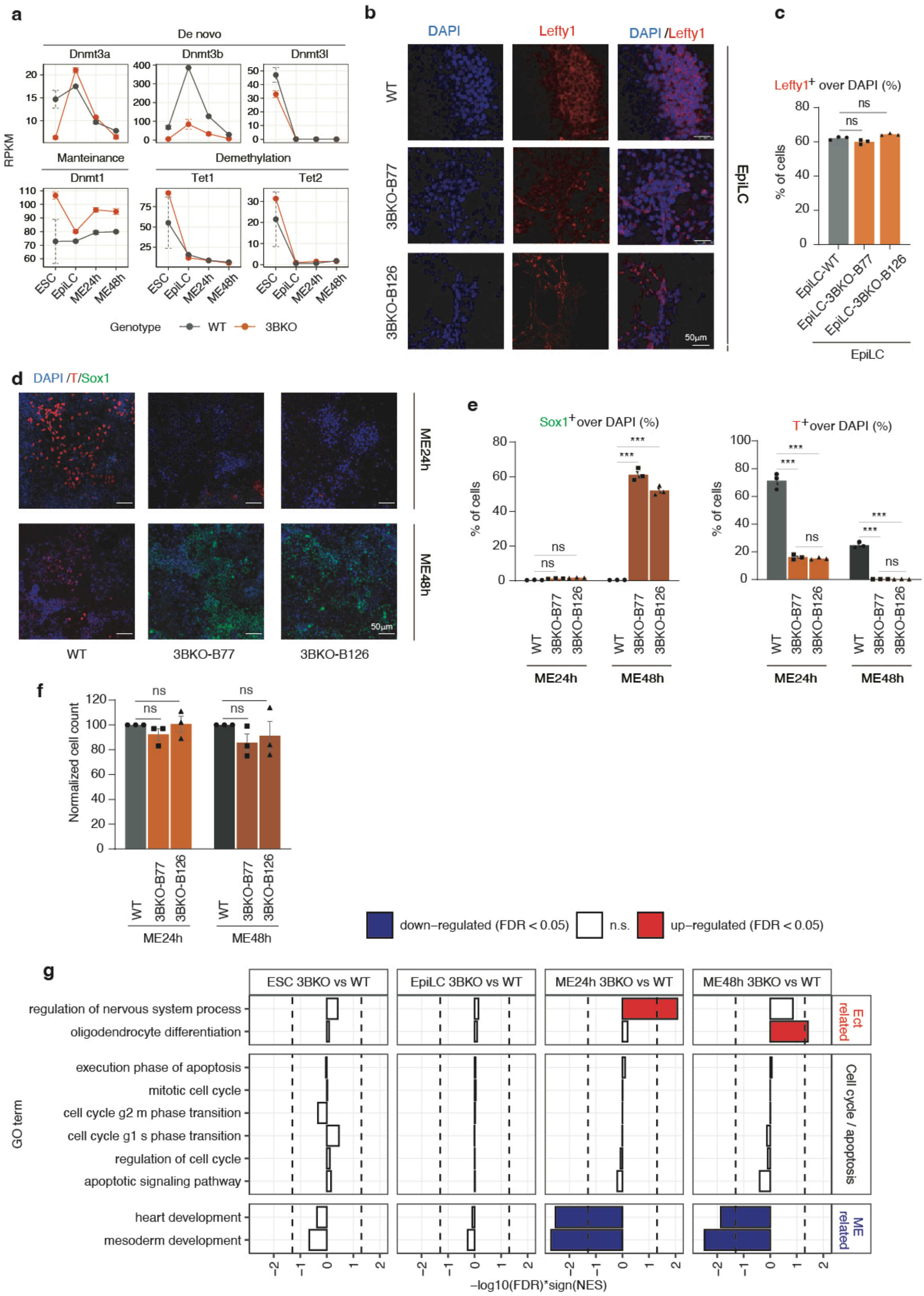
a
De novo
Dnmt3a
Dnmt3b
Dnmt3l
RPKM
Manteinance
Dnmt1
Demethylation
Tet1
Tet2
ESC
EpiLC
ME24h
ME48h
Genotype
WT
3BKO
b
DAPI
Lefty1
DAPI /Lefty1
WT
3BKO-B77
3BKO-B126
EpiLC
50µm
c
Lefty1+ over DAPI (%)
% of cells
ns
EpiLC-WT
EpiLC-3BKO-B77
EpiLC-3BKO-B126
EpiLC
d
DAPI /T/Sox1
ME24h
ME48h
WT
3BKO-B77
3BKO-B126
50µm
e
Sox1+over DAPI (%)
T+over DAPI (%)
% of cells
ns
***
WT
3BKO-B77
3BKO-B126
ME24h
ME48h
f
Normalized cell count
ns
WT
3BKO-B77
3BKO-B126
ME24h
ME48h
g
down–regulated (FDR < 0.05)
n.s.
up–regulated (FDR < 0.05)
ESC 3BKO vs WT
EpiLC 3BKO vs WT
ME24h 3BKO vs WT
ME48h 3BKO vs WT
GO term
regulation of nervous system process
oligodendrocyte differentiation
execution phase of apoptosis
mitotic cell cycle
cell cycle g2 m phase transition
cell cycle g1 s phase transition
regulation of cell cycle
apoptotic signaling pathway
heart development
mesoderm development
Ect related
Cell cycle / apoptosis
ME related
–log10(FDR)*sign(NES)

Supplementary Fig. 2

a, Gene expression levels from RNA-seq of DNA methyltransferases (*Dnmt1*, *Dnmt3a*, *Dnmt3b*, *Dnmt3l*) and methylcytosine dioxygenases (*Tet1*, *Tet2*) during the ESC-EpiLC-ME differentiation time course. Dots represent normalised RPKM values, averaged by replicates/condition (n=2 biological replicates for each genotype or clone at each time point). Error bars represent standard errors.

b, Representative IF images of WT and 3BKO (two independent clones, B126 and B77) EpiLCs stained with the primed pluripotency marker Lefty1 and counterstained with DAPI. Representative images of three independent experiments are shown. Scale bars 50 μm. Source data are provided as a Source Data file.

c, Quantification of Lefty1^{+} cells as percentages of Lefty1 over DAPI, for both WT and 3BKO cells, showing no significant differences in Lefty1^{+} cells at the EpiLC stage (ns: not significant, ANOVA test). Bars indicate mean±SEM of n=3 independent experiments for each genotype or clone, shown as dots). Error bars represent standard errors.

d, Representative IF images of WT and 3BKO (two independent clones, B77 and B126) ME24h and ME48h cells stained with the mesoderm marker (T) and neuro-ectoderm marker (Sox1), all counterstained with DAPI. The pictures show a stronger signal for T in WT with respect to 3BKO cells and a stronger signal for Sox1 in 3BKO with respect to WT cells. Representative images of three independent experiments are shown. Scale bars 50 μm. Source data are provided as a Source Data file.

e, Quantification of Sox1^{+} and T^{+} cells, shown as % of Sox1 over DAPI (left) and T over DAPI (right), for both WT and 3BKO cells. (*** = $p<0.001$, ANOVA test). Bars indicate mean±SEM of n=3 independent experiments for each genotype or clone at each time point, shown as dots). Error bars represent standard errors.

f, Cell number quantification of WT and 3BKO cells in ME24h and ME48h showing no significant growth reduction of knockout cells (ns: not significant, ANOVA test) Bars indicate mean±SEM of n=3 independent experiments for each genotype or clone at each time point, shown as dots). Error bars represent standard errors.

g, Barplot showing results of GSEA (https://doi.org/10.1073/pnas.0506580102) analysis comparing 3BKO and WT cells at the indicated differentiation stages. The -log10 of False Discovery Rate (FDR) multiplied by the sign of the normalized enrichment score (NES) is reported in the x axis. This analysis shows no significant differences between the two conditions in gene sets related to cell cycle and apoptosis, while 3BKO cells show increased expression of gene sets related to ectoderm and downregulation of gene sets related to meso-endoderm.

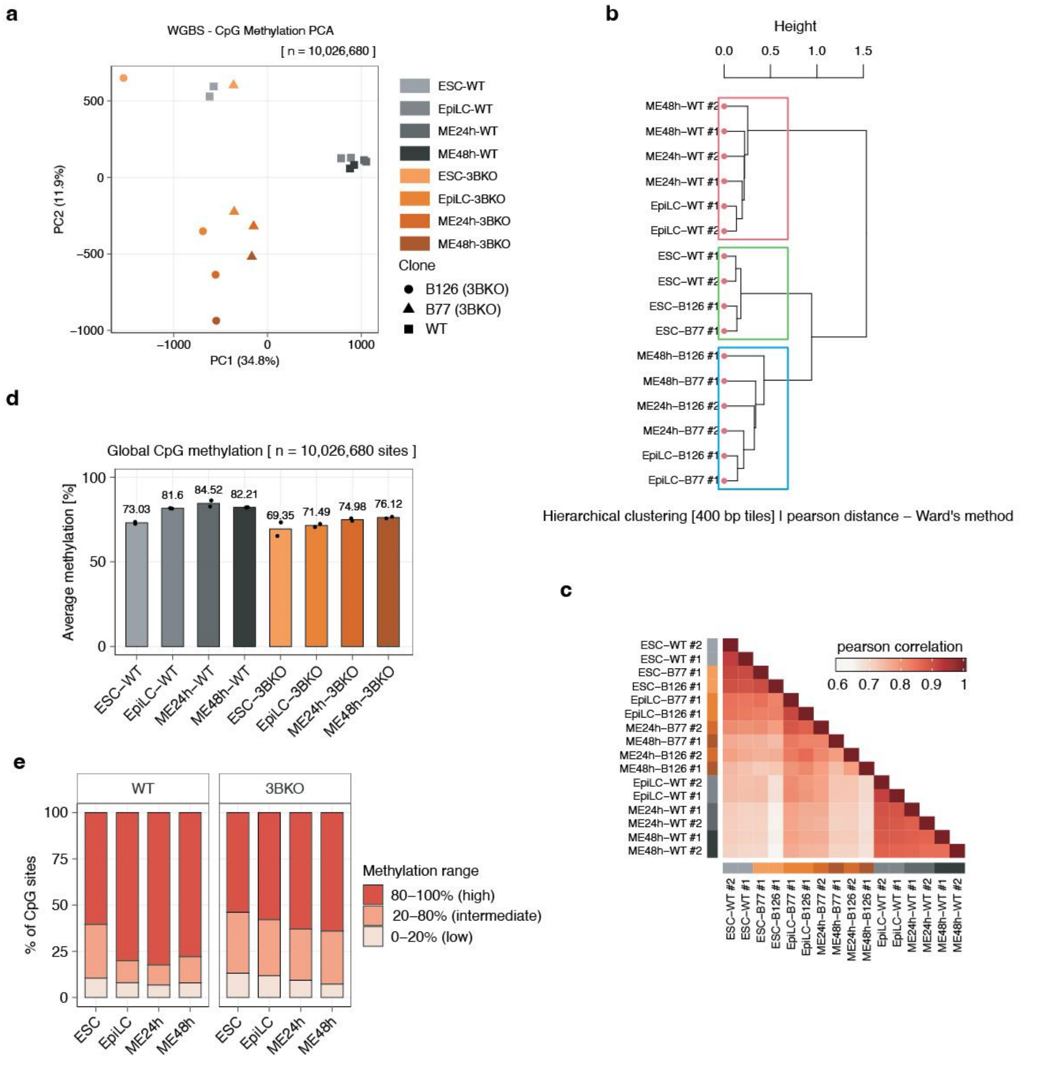

Supplementary Fig. 3

a, PCA of the DNA methylome profiles obtained by WGBS, performed at the single CpG resolution.

b, Hierarchical clustering of the DNA methylome profiles obtained by WGBS, using averaged DNAme scores in 400bp windows. The analysis was performed using Pearson correlation distance and Ward's method.

c, Heatmap showing pairwise Pearson correlation coefficients between the analyzed WGBS samples, calculated using averaged DNAme scores in 400bp windows.

d, Barplot showing the global average CpG methylation levels in each sample group. The dots represent the average global levels for each sample, the bars indicate the averaged value between replicates.

e, Stacked barplot showing distribution of genome-wide CpG methylation levels in each sample group, measured at individual CpG sites.

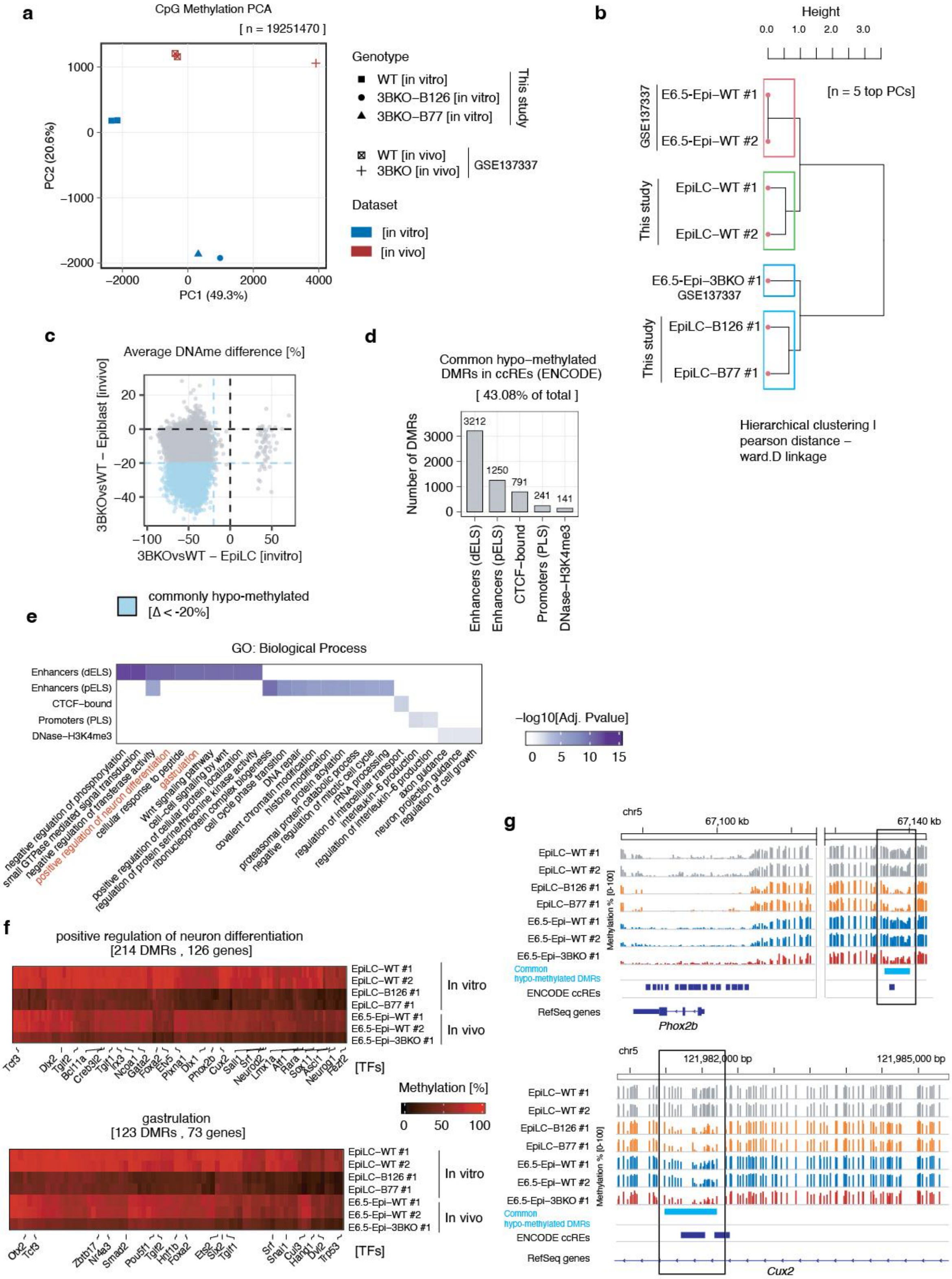
a
CpG Methylation PCA
[n = 19251470]
PC1 (49.3%)
PC2 (20.6%)
Genotype
WT [in vitro]
3BKO–B126 [in vitro]
3BKO–B77 [in vitro]
This study
WT [in vivo]
3BKO [in vivo]
GSE137337
Dataset
[in vitro]
[in vivo]
b
Height
[n = 5 top PCs]
E6.5-Epi–WT #1
E6.5-Epi–WT #2
EpiLC–WT #1
EpiLC–WT #2
E6.5-Epi–3BKO #1
EpiLC–B126 #1
EpiLC–B77 #1
Hierarchical clustering I
pearson distance –
ward.D linkage
c
Average DNAme difference [%]
3BKOvsWT – Epiblast [invivo]
3BKOvsWT – EpiLC [invitro]
commonly hypo-methylated
[Δ < -20%]
d
Common hypo–methylated
DMRs in ccREs (ENCODE)
[43.08% of total]
Number of DMRs
3212
1250
791
241
141
Enhancers (dELS)
Enhancers (pELS)
CTCF–bound
Promoters (PLS)
DNase–H3K4me3
e
GO: Biological Process
–log10[Adj. Pvalue]
negative regulation of phosphorylation
small GTPase mediated signal transduction
negative regulation of transferase activity
positive regulation of neuron differentiation
cellular response to peptide
gastrulation
Wnt signaling pathway
cell–cell signaling by wnt
positive regulation of cellular protein localization
regulation of protein serine/threonine kinase activity
ribonucleoprotein complex biogenesis
cell cycle phase transition
DNA repair
covalent chromatin modification
histone modification
protein acylation
proteasomal protein catabolic process
negative regulation of mitotic cell cycle
rRNA processing
regulation of intracellular transport
interleukin–6 production
regulation of interleukin–6 production
axon guidance
neuron projection guidance
regulation of cell growth
f
positive regulation of neuron differentiation
[214 DMRs , 126 genes]
In vitro
In vivo
[TFs]
Methylation [%]
gastrulation
[123 DMRs , 73 genes]
g
chr5
67,100 kb
67,140 kb
Methylation % [0-100]
Common hypo-methylated DMRs
ENCODE ccREs
RefSeq genes
Phox2b
121,982,000 bp
121,985,000 bp
Cux2

Supplementary Fig. 4

a, PCA of the DNA methylome profiles obtained by WGBS, performed at the single CpG resolution, for WT and 3BKO in vitro EpiLCs and in vivo epiblast from E6.5 mouse embryos[2]. Genotype (3BKO, WT) is driving substantial variation on PC1 (i.e. the main direction of variability), while *in vivo* / *in vitro* samples are separated in PC2.
b, Hierarchical clustering of the DNA methylome profiles obtained by WGBS, using the top 5 PCs. WT and 3BKO samples are driving the separation between the two main branches, with *in vitro* WT EpiLCs clustering with *in vivo* WT epiblasts and *in vitro* 3BKO EpiLCs clustering with *in vivo* 3BKO epiblasts. The analysis was performed using Pearson correlation distance and Ward's method.
c, Scatterplot showing the average DNAme difference (as %) at the DMRs identified *in vitro* (EpiLC) between 3BKO and WT, for *in vitro* (EpiLC, x-axis) and *in vivo* (Epi, y-axis) cells. The comparative analysis showed that 40.3% of *in vitro* DMRs are consistently hypomethylated *in vivo* (delta <-20%). The commonly hypomethylated regions are depicted in blue.
d, Barplot showing the number of commonly hypomethylated DMRs between 3BKO and WT overlapping annotated candidate cis-regulatory elements (ccREs) retrieved from ENCODE SCREEN database[3] for mouse mm10. 43.08% of these DMRs overlaps with ccREs, with highest overlap in the elements annotated as distal Enhancer-Like Signature (dELS).
e, Heatmap showing adjusted p-values (hypergeometric test as implemented in the ClusterProfiler package[4] for GO terms of enriched biological processes in each of the five ccREs reported in **d**. The analysis revealed significant enrichment of developmental gene sets (e.g. gastrulation) involved in neuronal differentiation (e.g. positive regulation of neuronal differentiation) in the genes associated with commonly hypomethylated distal enhancers.
f, WGBS heatmap visualising the average DNAme (as %) levels of the DMRs associated to the genes in the GO terms positive regulation of neuron differentiation (top) and gastrulation (bottom), highlighted in red on panel **e**, for both *in vitro* and *in vivo* samples. The heatmaps show consistent reduction of DNAme levels in 3BKO samples compared to their WT counterparts in both *in vitro* EpiLCs and *in vivo* epiblast cells.
g, Genome browser views showing the WGBS signal profiles of WT and 3BKO *in vitro* EpiLC and *in vivo* epiblast cells on the genomic loci surrounding two representative genes - *Cux2* and *Phox2b* - involved in neural differentiation. The commonly hypomethylated regions (depicted in blue and indicated in the rectangles) overlap annotated ENCODE ccREs.

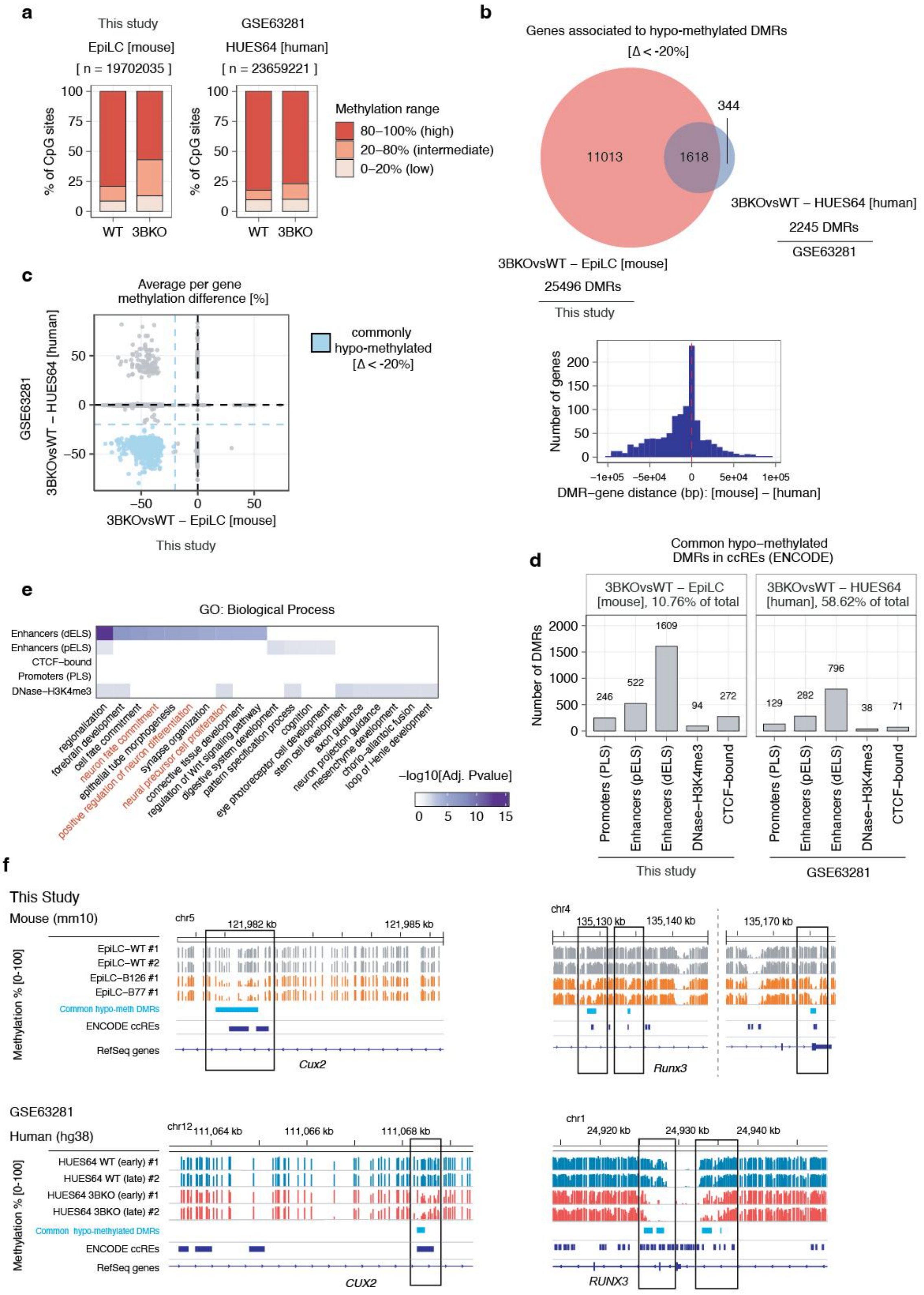
a
This study
EpiLC [mouse]
[n = 19702035]
GSE63281
HUES64 [human]
[n = 23659221]
% of CpG sites
WT 3BKO
Methylation range
80–100% (high)
20–80% (intermediate)
0–20% (low)
b
Genes associated to hypo-methylated DMRs
[Δ < -20%]
11013
1618
344
3BKOvsWT – HUES64 [human]
2245 DMRs
GSE63281
3BKOvsWT – EpiLC [mouse]
25496 DMRs
This study
Number of genes
DMR–gene distance (bp): [mouse] – [human]
c
Average per gene methylation difference [%]
commonly hypo-methylated [Δ < -20%]
GSE63281
3BKOvsWT – HUES64 [human]
3BKOvsWT – EpiLC [mouse]
This study
d
Common hypo–methylated DMRs in ccREs (ENCODE)
3BKOvsWT – EpiLC [mouse], 10.76% of total
3BKOvsWT – HUES64 [human], 58.62% of total
Number of DMRs
246
522
1609
94
272
129
282
796
38
71
Promoters (PLS)
Enhancers (pELS)
Enhancers (dELS)
DNase–H3K4me3
CTCF–bound
This study
GSE63281
e
GO: Biological Process
Enhancers (dELS)
Enhancers (pELS)
CTCF–bound
Promoters (PLS)
DNase–H3K4me3
regionalization
forebrain development
cell fate commitment
neuron fate commitment
epithelial tube morphogenesis
positive regulation of neuron differentiation
synapse organization
neural precursor cell proliferation
connective tissue development
regulation of Wnt signaling pathway
digestive system development
pattern specification process
eye photoreceptor cell development
cognition
stem cell development
axon guidance
neuron projection guidance
mesenchyme development
chorio–allantoic fusion
loop of Henle development
−log10[Adj. Pvalue]
f
This Study
Mouse (mm10)
Methylation % [0-100]
EpiLC–WT #1
EpiLC–WT #2
EpiLC–B126 #1
EpiLC–B77 #1
Common hypo-meth DMRs
ENCODE ccREs
RefSeq genes
chr5
121,982 kb
121,985 kb
Cux2
chr4
135,130 kb
135,140 kb
135,170 kb
Runx3
GSE63281
Human (hg38)
HUES64 WT (early) #1
HUES64 WT (late) #2
HUES64 3BKO (early) #1
HUES64 3BKO (late) #2
Common hypo-methylated DMRs
chr12
111,064 kb
111,066 kb
111,068 kb
CUX2
chr1
24,920 kb
24,930 kb
24,940 kb
RUNX3

Supplementary Fig.5

a, Stacked barplot showing distribution of genome-wide CpG methylation levels in WT and 3BKO cells in our mouse EpiLCs and in human HUES64 cells, measured at individual CpG sites. The WT HUES64 cells showed higher numbers of highly methylated CpG with respect to mouse EpiLCs. The mouse 3BKO in EpiLCs showed a stronger reduction of DNAme levels as compared to WT with respect to the human 3BKO HUES64.

b, (top) Venn diagram showing the overlap of genes associated to a 3BKO hypomethylated DMRs between mouse (left) and human (right). Differential methylation analysis between 3BKO and WT samples identified 25,496 DMRs in mouse cells and 2,245 DMRs in human cells. After linking the identified DMRs to genes and associating mouse genes to their respective human orthologs, the comparative analysis showed that 1618 genes having at least one associated hypomethylated region (within 100kb from the TSS) in human had also at least one associated hypomethylated region in mouse (methylation difference <-20%). (bottom) Histogram showing difference of the DMR-genes distances between mouse and human commonly hypomethylated genes.

c, Scatterplot showing the average per gene DNAme difference (as %) for the human-mouse ortholog genes associated with at least one DMR between 3BKO and WT cells, either in mouse EpiLCs (x-axis) or in human HUES64 cells (y-axis). The commonly hypomethylated genes are depicted in blue.

d, Barplot showing the number of commonly hypomethylated DMRs between 3BKO and WT overlapping annotated candidate cis-regulatory elements (ccREs) retrieved from ENCODE SCREEN database[3] for mouse mm10 and human hg38. 17.36% (in mouse) and 88.86% (in human) overlap with ccREs, with highest overlap in the elements annotated as distal Enhancer-Like Signature (dELS).

e, Heatmap showing adjusted p-values (hypergeometric test as implemented in the ClusterProfiler package[4] for GO terms of enriched biological processes in each of the five ccREs reported in **d**. The analysis revealed significant enrichment of developmental gene sets involved in neuronal differentiation (e.g. positive regulation of neuronal differentiation, neuron fate commitment, neural precursor cell proliferation) in the genes associated with commonly hypomethylated distal enhancers.

f, Genome browser views showing the WGBS signal profiles of *in vitro* WT and 3BKO mouse EpiLCs and human HUES64 cells on the genomic loci surrounding two representative genes - *Cux2* and *Runx3* - involved in neural differentiation. The commonly hypomethylated regions (depicted in blue and indicated in the rectangles) overlap annotated ENCODE ccREs.

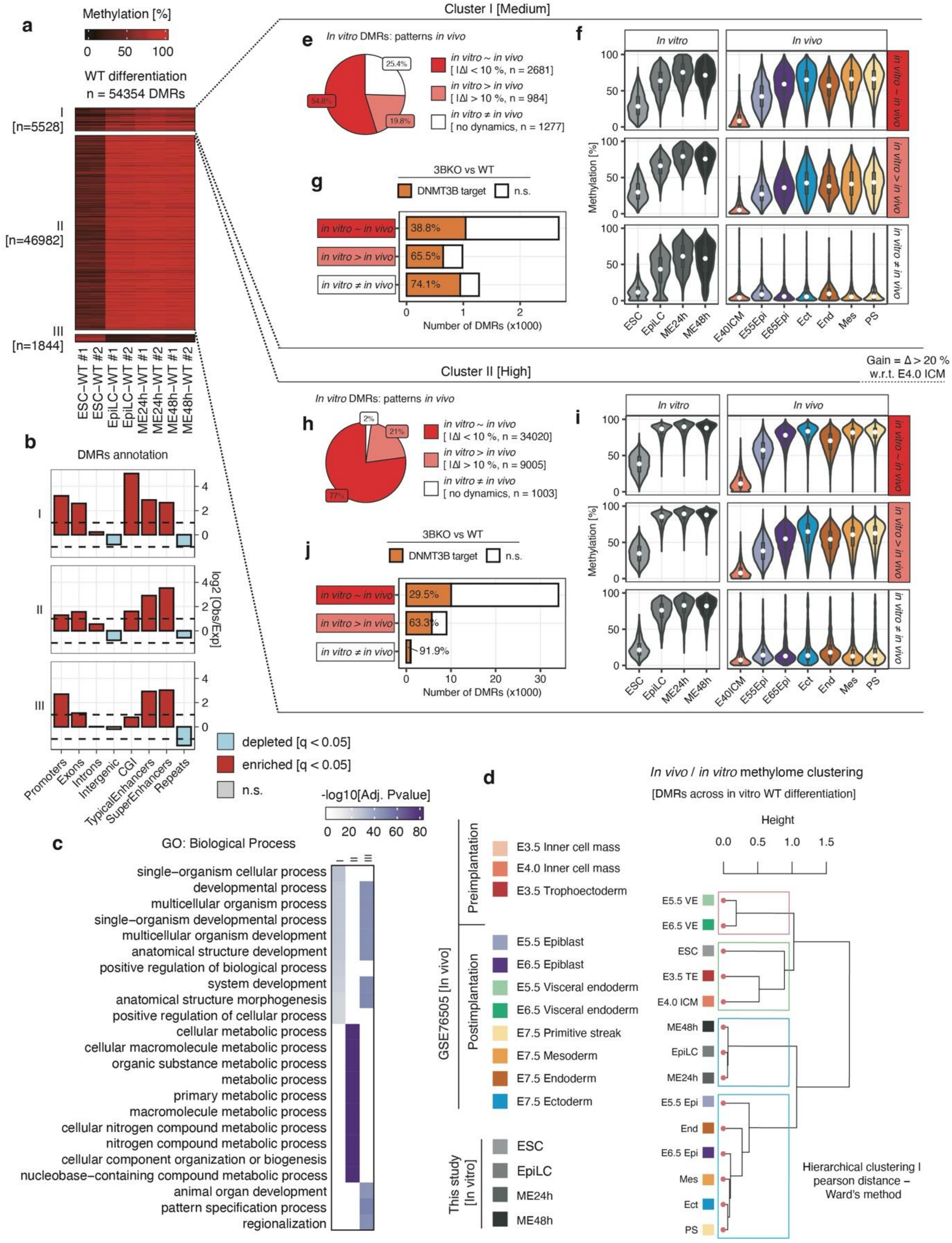

a
Methylation [%]
0 50 100
WT differentiation
n = 54354 DMRs
I [n=5528]
II [n=46982]
III [n=1844]
ESC−WT #1
ESC−WT #2
EpiLC−WT #1
EpiLC−WT #2
ME24h−WT #1
ME24h−WT #2
ME48h−WT #1
ME48h−WT #2
b
DMRs annotation
log2 [Obs/Exp]
Promoters
Exons
Introns
Intergenic
CGI
TypicalEnhancers
SuperEnhancers
Repeats
depleted [q < 0.05]
enriched [q < 0.05]
n.s.
c
GO: Biological Process
-log10[Adj. Pvalue]
0 20 40 60 80
I II III
single−organism cellular process
developmental process
multicellular organism process
single−organism developmental process
multicellular organism development
anatomical structure development
positive regulation of biological process
system development
anatomical structure morphogenesis
positive regulation of cellular process
cellular metabolic process
cellular macromolecule metabolic process
organic substance metabolic process
metabolic process
primary metabolic process
macromolecule metabolic process
cellular nitrogen compound metabolic process
nitrogen compound metabolic process
cellular component organization or biogenesis
nucleobase−containing compound metabolic process
animal organ development
pattern specification process
regionalization
Cluster I [Medium]
e
In vitro DMRs: patterns in vivo
25.4%
54.8%
19.8%
in vitro ~ in vivo [|Δ| < 10 %, n = 2681]
in vitro > in vivo [|Δ| > 10 %, n = 984]
in vitro ≠ in vivo [no dynamics, n = 1277]
g
3BKO vs WT
DNMT3B target
n.s.
in vitro ~ in vivo
in vitro > in vivo
in vitro ≠ in vivo
38.8%
65.5%
74.1%
Number of DMRs (x1000)
f
In vitro
In vivo
Methylation [%]
ESC
EpiLC
ME24h
ME48h
E40ICM
E55Epi
E65Epi
Ect
End
Mes
PS
Gain = Δ > 20 %
w.r.t. E4.0 ICM
Cluster II [High]
h
In vitro DMRs: patterns in vivo
2%
21%
77%
in vitro ~ in vivo [|Δ| < 10 %, n = 34020]
in vitro > in vivo [|Δ| > 10 %, n = 9005]
in vitro ≠ in vivo [no dynamics, n = 1003]
j
3BKO vs WT
DNMT3B target
n.s.
29.5%
63.3%
91.9%
Number of DMRs (x1000)
i
In vitro
In vivo
Methylation [%]
d
In vivo / in vitro methylome clustering
[DMRs across in vitro WT differentiation]
Height
0.0 0.5 1.0 1.5
GSE76505 [In vivo]
Preimplantation
E3.5 Inner cell mass
E4.0 Inner cell mass
E3.5 Trophoectoderm
Postimplantation
E5.5 Epiblast
E6.5 Epiblast
E5.5 Visceral endoderm
E6.5 Visceral endoderm
E7.5 Primitive streak
E7.5 Mesoderm
E7.5 Endoderm
E7.5 Ectoderm
This study [In vitro]
ESC
EpiLC
ME24h
ME48h
E5.5 VE
E6.5 VE
ESC
E3.5 TE
E4.0 ICM
ME48h
EpiLC
ME24h
E5.5 Epi
End
E6.5 Epi
Mes
Ect
PS
Hierarchical clustering | pearson distance – Ward's method

Supplementary Fig. 6

a, (left) WGBS heatmap showing the DMRs arising during the ESC-EpiLC-ME differentiation in WT cells, clustered by K-means. Each cluster shows a distinct pattern of DNAme levels and dynamics, defined as Medium (I), High (II) and Demethylated (III). (right) Boxplot distributions of the DNAme levels (as %) for the CpG sites in each DMR cluster.

b, Annotation of DMRs to distinct genomic features, reported as (top) the log2-enrichment for each feature and (bottom) the percentage of DMRs overlapping each feature, calculated with the Genomic Association Test (GAT) software for each DMR cluster reported in **a**.

c, Heatmap showing GO terms for enriched biological processes in each DMR cluster reported in **a**. Gene set over-representation analysis was performed for genes associated with DMRs using hypergeometric tests as implemented in GREAT[5], correcting for multiple hypotheses.

d, Hierarchical clustering of WGBS data from the *in vitro* differentiation and *in vivo* embryonic tissues derived from pre- and post-implantation mouse embryos[6] performed on the DMRs arising over the in vitro WT differentiation. Pearson's correlation distance and Ward's method were employed to perform the analysis.

e, Pie chart showing the classification of i*n vitro* DMRs in comparison to DNAme levels in embryonic tissues from[4] in Cluster I (Medium). The DMRs were grouped as: *in vitro* ~ *in vivo* (red): DMRs that gain *de novo* DNAme both *in vitro* (between ESC and later stages) and *in vivo* (between ICM and later stages), reaching similar average levels of DNAme in the two systems (i.e. less than 10% difference). *in vitro* > *in vivo* (light red): DMRs that gain *de novo* DNAme both *in vitro* (between ESC and later stages) and *in vivo* (between ICM and later stages), but with higher DNAme levels *in vitro* with respect to *in vivo* (i.e. more than 10% difference). (white) *in vitro* ≠ *in vivo*: DMRs that gain *de novo* DNAme *in vitro* (between ESC and later stages) but have little dynamics *in vivo*, where they remain mostly hypomethylated.

f, Violin plots showing the DNAme levels distribution in each group for in vitro time points (average of n=2 biological replicates) and in vivo embryonic tissues in Cluster I (Medium). White dots indicate median, box indicates the interquartile range (IQR) and whiskers denote the 1.5 × IQR.

g, Barplot showing the percentage of DMT3B-dependent DMRs in each group in Cluster I (Medium).

h, **i, j**. Pie chart (h), violin plots (i) and barplot (j) as in d,e,f for Cluster II (High).

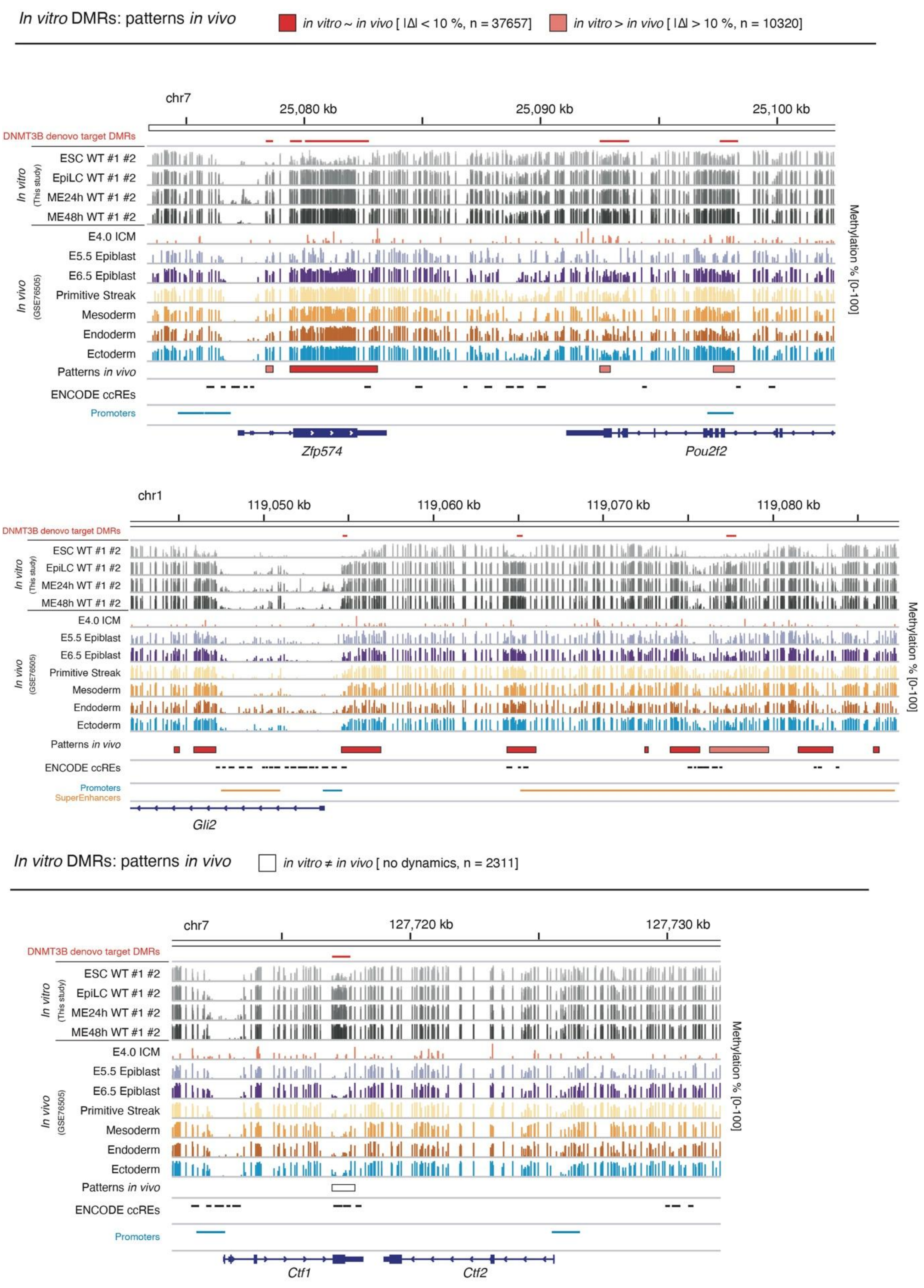

Supplementary Fig. 7

Genome browser views showing WGBS signal profiles representative of the *in vitro* and *in vivo*[6] dynamics across differentiation stages. Annotations for regulatory regions (promoters/typical and super enhancers, as defined by ChIP-seq data), CpG islands and ENCODE candidate *cis*-regulatory elements (ccREs) for mouse mm10[3] are also reported.

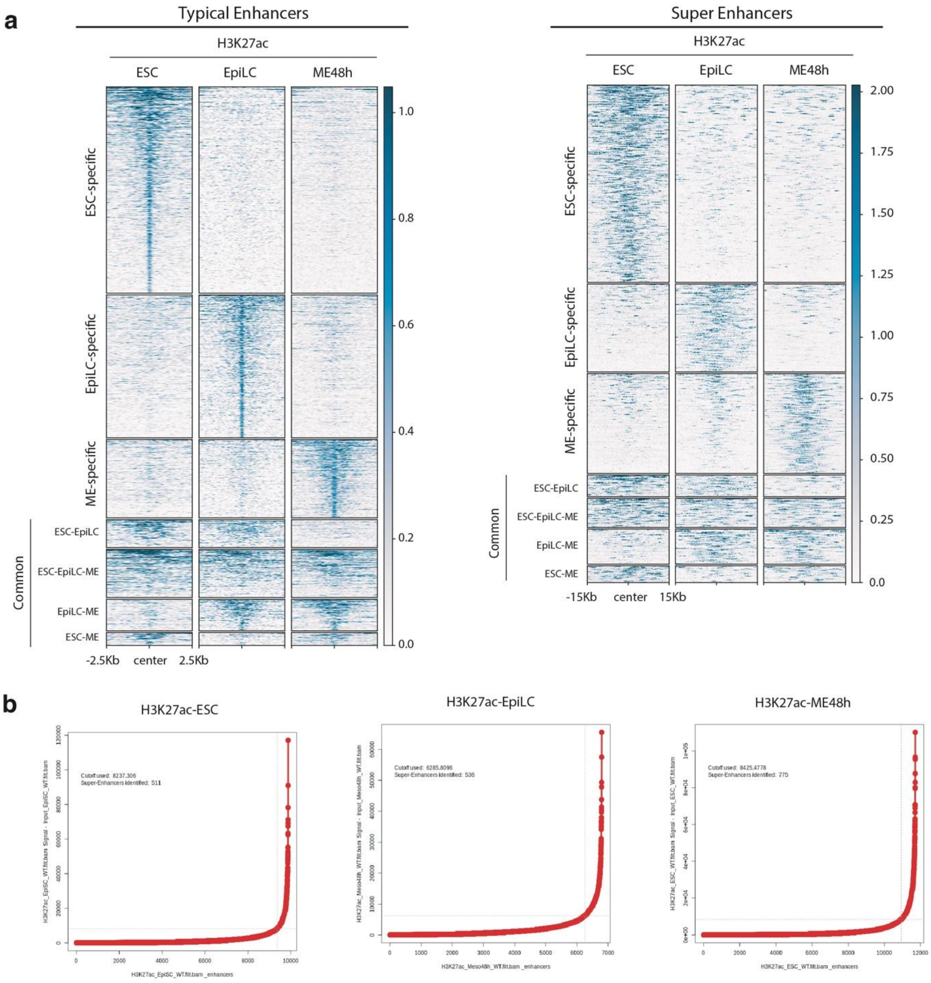

Supplementary Fig. 8

a, Heatmaps showing H3K27ac ChIP-seq signal for WT cells over the differentiation time course, clustered in stage-specific or shared-by-stage regions, for typical (left) and super enhancer (right) regions.

b, Ranking plots of H3K27ac ChIP-seq signals for WT cells at each time point of differentiation obtained from ROSE. Inflection points of the curves represent the cut-off for super enhancer definition.

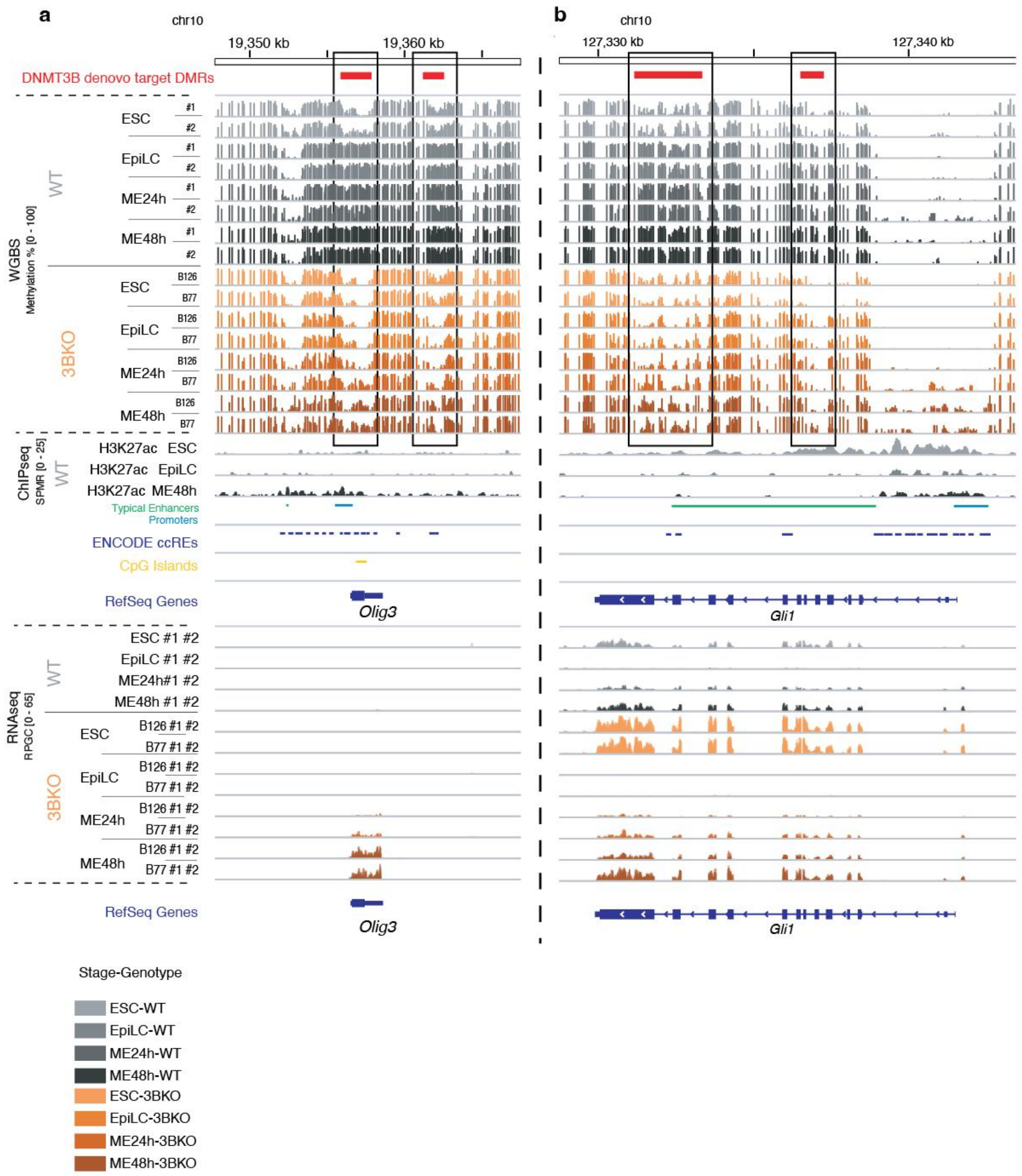

Supplementary Fig. 9

Genome browser views showing the WGBS, ChIP-seq and RNA-seq signal profiles across differentiation (ESC-EpiLC-ME) for WT and 3BKO cells on the **a**, *Olig3* and **b**, *Gli1* gene loci, which are two representative TFs involved in neuro-ectodermal differentiation regulated by DNMT3B. The identified DNMT3B *de novo* target DMRs (depicted in red and indicated in the rectangles) overlap with gene promoters and enhancers (identified by H3K27ac ChIP-seq signals and previously annotated in[7]). Annotations for regulatory regions (promoters/typical and super enhancers, as defined by ChIP-seq data), CpG islands and ENCODE candidate *cis*-regulatory elements (ccREs) for mouse mm10[3] are also reported.

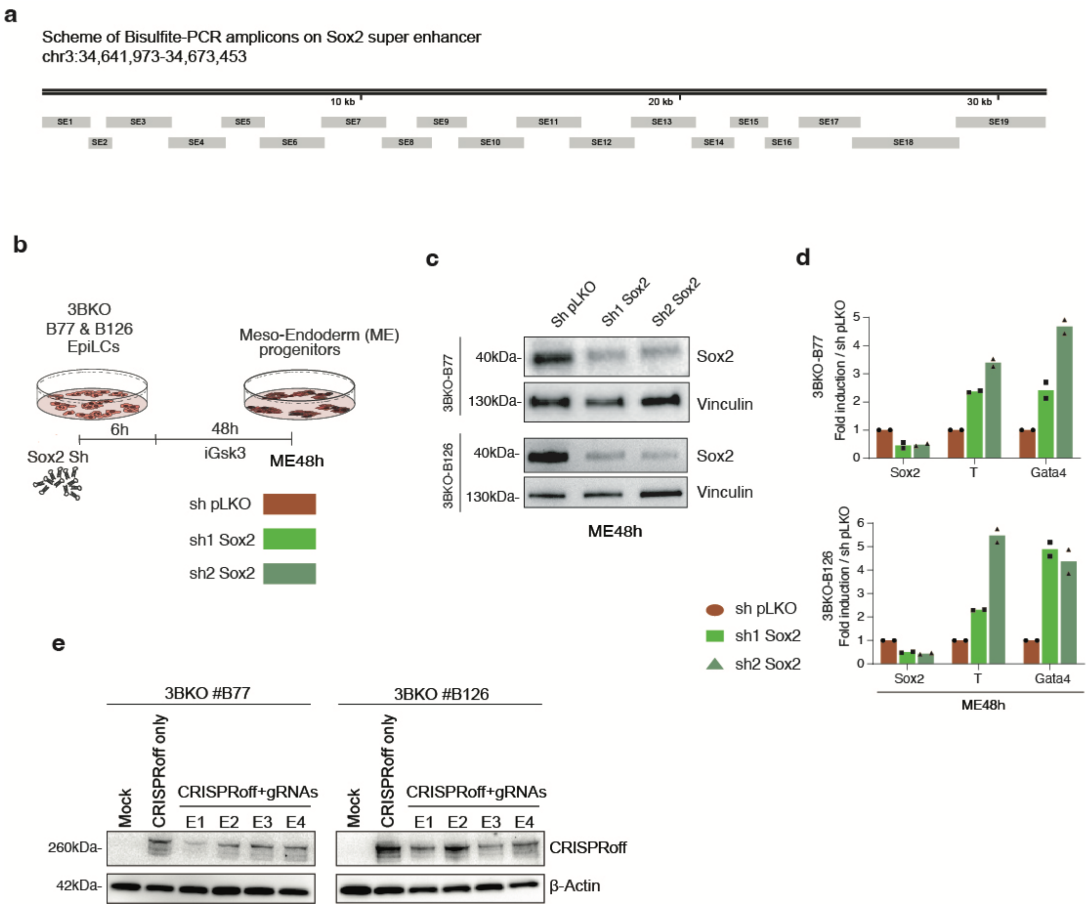

Supplementary Fig. 10

a, Scheme of Bisulfite-PCR amplicons on *Sox2* super enhancer (chr3:34,641,973-34,673,453).

b, Schematic of *Sox2* silencing experiment during meso-endoderm differentiation.

c, WB of 3BKO ME48h cells (two independent clones, B77 and B126) silenced with two different shRNAs against Sox2 (Sh-Scramble is used as negative control), showing substantial reduction of Sox2 expression. Vinculin serves as loading control. Representative of two independent experiments. Uncropped gels are provided in Supplementary Fig. 11.

d, RT-qPCR analyses for ectoderm (*Sox2*), mesoderm (*T*) and endoderm (*Gata4*) genes in *Sox2* silenced and control cells in each 3BKO clone, showing significant reduction of *Sox2* expression and a significant induction of *T* and *Gata4* expression upon *Sox2* depletion. Bars indicate the mean of two independent experiments shown as dots. Expression was normalised to sh pLKO control.

e, Western blot of CRISPRoff protein expression in two 3BKO clones measured at 24 hours post-transfection without or with three gRNAs targeting Sox2 super enhancer elements (E1-E4). β-Actin was used as a loading control. Representative of two independent experiments. Uncropped gels are provided in Supplementary Fig. 11.

Source data_uncropped western blot gels

Figure 2d

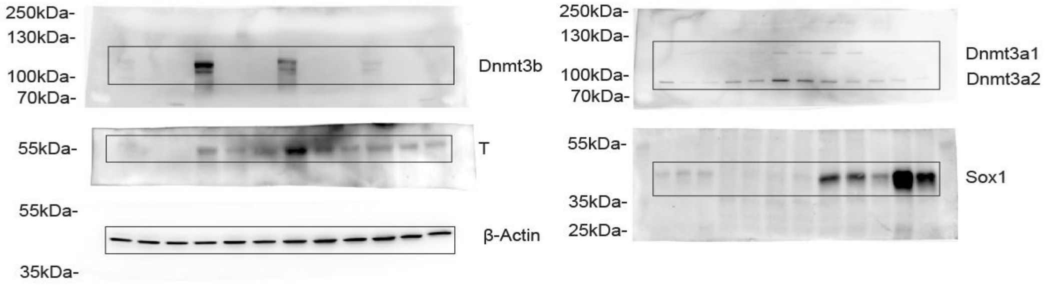

Figure 3a

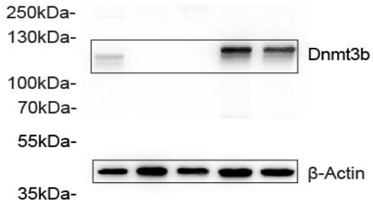

Figure S9e

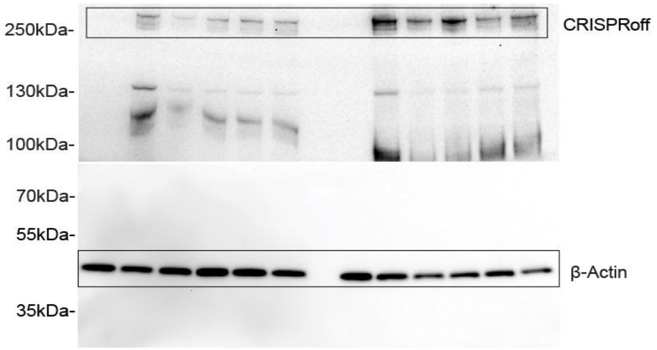

Figure S9c

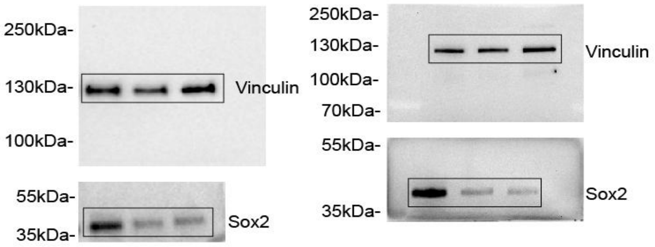

Supplementary Fig. 11.

Original uncropped western blot images used in this study. Rectangles indicate the cropped regions.

References

1. Cao, J. *et al.* The single-cell transcriptional landscape of mammalian organogenesis. *Nature* 566, 496–502 (2019).

2. Grosswendt, S. *et al.* Epigenetic regulator function through mouse gastrulation. *Nature* 584, 102–108 (2020).

3. Consortium, T. E. P. *et al.* Expanded encyclopaedias of DNA elements in the human and mouse genomes. *Nature* 583, 699–710 (2020).

4.Yu, G. et al. clusterProfiler: an R Package for Comparing Biological Themes Among Gene Clusters. *Omics*: *A Journal of Integrative Biology*, 16, 284-287 (2012).

5. McLean, C. Y. *et al.* GREAT improves functional interpretation of cis-regulatory regions. *Nat Biotechnol* 28, 495–501 (2010)

6. Zhang, Y. *et al.* Dynamic epigenomic landscapes during early lineage specification in mouse embryos. *Nat Genet* 50, 96–105 (2017).

7. Zhou, H. Y. *et al.* A Sox2 distal enhancer cluster regulates embryonic stem cell differentiation potential. *Gene Dev* 28, 2699–2711 (2014).
