# Supplementary material for: DNMT3B supports meso-endoderm differentiation from mouse embryonic stem cells
Source: Nat Commun. 2023 Jan 23;14:367. doi: 10.1038/s41467-023-35938-x (PMC9871038; doi:10.1038/s41467-023-35938-x)
Supplement: Supplementary file 2 — Description of Additional Supplementary Files [file 41467_2023_35938_MOESM2_ESM.pdf]

Description of Additional Supplementary Files:

Supplementary Data 1. scRNA-seq clusters markers. Marker genes identified for each cell cluster by differential expression analysis, using the Logistic Regression (LR) approach implemented in the FindAllMarkers function from the Seurat package. Reported genes are ordered by p-value. Significance thresholds: p-value less than 0.01.

Supplementary Data 2. Trajectory-dependent gene expression analysis. Results of the graph-autocorrelation analysis to find genes that vary as a function of pseudotime (graph_test function in Monocle3). Genes are grouped according to the branch in which they show significant variation (i.e., Epi-to-Ect specific, Epi-to-ME specific, commonly regulated). Significance thresholds: q-value less than 0.01.

Supplementary Data 3. Gene expression profiles of WT and 3BKO ESC-EpiLC-ME differentiation. RNA-seq analysis of WT and 3BKO cells in ESC-EpiLC-MesoEndoderm (ME) differentiation time course. The first panel (i.e. 'edgeR') reports differentially expressed genes at any stage/condition (edgeR anova-like test for any difference, logFC greater or equal to 1.5 and FDR less than or equal to 0.001), used for clustering the gene expression profiles. Second panel (i.e. 'K-means clustering') reports the result of gene expression profile clustering by K-means, together with the normalized gene expression levels for each gene in each sample group (averaged between replicates and 3BKO clones). The remaining panels report the differentially expressed genes between 3BKO and WT cells at each time point of the differentiation time course (i.e ESC, EpiLC, ME24h, ME48h, logFC greater or equal to 1 and FDR less than or equal to 0.05).

Supplementary Data 4. Gene ontology analysis of gene expression clusters. Results of gene ontology analysis (adjusted P-value less than or equal to 0.05) for each of the four gene clusters identified by K-means.

Supplementary Data 5. RNA-seq analysis of DNMT3B ectopic expression in 3BKO cells. RNA-seq analysis of 3BKO and 3BKO ectopically expressing DNMT3B cells in EpiLC-MesoEndoderm (ME) differentiation time course. The first three panels report the differentially expressed genes between 3BKO+3B and 3BKO cells at each time point of the differentiation time course (EpiLC, ME24h, ME48h, absolute logFC greater or equal to 0.5 and FDR less than or equal to 0.05). The last panel reports the list of rescued genes among the differentially regulated genes between 3BKO and WT cells across differentiation.

Supplementary Data 6. Differentially methylated regions (DMRs) over the ESC-EpiLC-ME differentiation. Lists of the DMRs arising over the differentiation time course for WT cells (first panel), and those showing impaired de novo DNAme in 3BKO cells (second panel). For each DMR, the average methylation level in each sample, the DMR cluster (retrieved by K-means from the WT

differentiation). the association to genes, the overlap with regulatory regions (annotated in our system and/or as ccREs in ENCODE SCREEN) and other epigenomic data are reported.

Supplementary Data 7. Integrated analysis. Integrated RNA-seq/WGBS/ChIP-seq analysis results. For each of the 615 DNMT3B direct target genes, the associated DMRs are reported (mm10 genomic coordinates, distance from TSS, type of regulatory region), their average expression and DNAme levels, and gene ontology enrichment results for each gene cluster (early/mid/late induced).

Supplementary Data 8. Lists of oligonucleotides, gRNA sequences and antibodies used in this study. List of oligonucletides used for the RT-qPCR experiments, shRNA cloning and BSAS analysis. gRNA sequences used in the CRISPRoff experiment. List of antobodies used in this study.
