# Supplementary material for: DNMT3B supports meso-endoderm differentiation from mouse embryonic stem cells
Source: Nat Commun. 2023 Jan 23;14:367. doi: 10.1038/s41467-023-35938-x (PMC9871038; doi:10.1038/s41467-023-35938-x)
Supplement: Supplementary file 11 — Reporting Summary [file 41467_2023_35938_MOESM11_ESM.pdf]

nature portfolio

Corresponding author(s): Salvatore Oliviero

Last updated by author(s): Dec 23, 2022

Reporting Summary

Nature Portfolio wishes to improve the reproducibility of the work that we publish. This form provides structure for consistency and transparency in reporting. For further information on Nature Portfolio policies, see our Editorial Policies and the Editorial Policy Checklist.

Statistics

For all statistical analyses, confirm that the following items are present in the figure legend, table legend, main text, or Methods section.

n/a	Confirmed	
☐	☒	The exact sample size (*n*) for each experimental group/condition, given as a discrete number and unit of measurement
☐	☒	A statement on whether measurements were taken from distinct samples or whether the same sample was measured repeatedly
☐	☒	The statistical test(s) used AND whether they are one- or two-sided<br>*Only common tests should be described solely by name; describe more complex techniques in the Methods section.*
☐	☒	A description of all covariates tested
☐	☒	A description of any assumptions or corrections, such as tests of normality and adjustment for multiple comparisons
☐	☒	A full description of the statistical parameters including central tendency (e.g. means) or other basic estimates (e.g. regression coefficient) AND variation (e.g. standard deviation) or associated estimates of uncertainty (e.g. confidence intervals)
☐	☒	For null hypothesis testing, the test statistic (e.g. *F*, *t*, *r*) with confidence intervals, effect sizes, degrees of freedom and *P* value noted<br>*Give P values as exact values whenever suitable.*
☐	☒	For Bayesian analysis, information on the choice of priors and Markov chain Monte Carlo settings
☒	☐	For hierarchical and complex designs, identification of the appropriate level for tests and full reporting of outcomes
☐	☒	Estimates of effect sizes (e.g. Cohen's *d*, Pearson's *r*), indicating how they were calculated

Our web collection on statistics for biologists contains articles on many of the points above.

Software and code

Policy information about availability of computer code

Data collection	SRA-toolkit
Data analysis	Bowtie2; Bismark; STAR; R; Python; SAMtools; BEDtools;

For manuscripts utilizing custom algorithms or software that are central to the research but not yet described in published literature, software must be made available to editors and reviewers. We strongly encourage code deposition in a community repository (e.g. GitHub). See the Nature Portfolio guidelines for submitting code & software for further information.

Data

Policy information about availability of data

All manuscripts must include a data availability statement. This statement should provide the following information, where applicable:

- Accession codes, unique identifiers, or web links for publicly available datasets
- A description of any restrictions on data availability
- For clinical datasets or third party data, please ensure that the statement adheres to our policy

GSE168415
https://www.ncbi.nlm.nih.gov/geo/query/acc.cgi?acc=GSE168415

Human research participants

Policy information about studies involving human research participants and Sex and Gender in Research.

Reporting on sex and gender	does not apply
Population characteristics	does not apply
Recruitment	does not apply
Ethics oversight	does not apply

Note that full information on the approval of the study protocol must also be provided in the manuscript.

Field-specific reporting

Please select the one below that is the best fit for your research. If you are not sure, read the appropriate sections before making your selection.

☒ Life sciences ☐ Behavioural & social sciences ☐ Ecological, evolutionary & environmental sciences

For a reference copy of the document with all sections, see nature.com/documents/nr-reporting-summary-flat.pdf

Life sciences study design

All studies must disclose on these points even when the disclosure is negative.

Sample size	does not apply
Data exclusions	does not apply
Replication	Per performed at least 3 replications for each experiment.
Randomization	does not apply
Blinding	does not apply

Reporting for specific materials, systems and methods

We require information from authors about some types of materials, experimental systems and methods used in many studies. Here, indicate whether each material, system or method listed is relevant to your study. If you are not sure if a list item applies to your research, read the appropriate section before selecting a response.

Materials & experimental systems

n/a	Involved in the study	
☐	☒	Antibodies
☐	☒	Eukaryotic cell lines
☒	☐	Palaeontology and archaeology
☒	☐	Animals and other organisms
☒	☐	Clinical data
☒	☐	Dual use research of concern

Methods

n/a	Involved in the study	
☐	☒	ChIP-seq
☒	☐	Flow cytometry
☒	☐	MRI-based neuroimaging

Antibodies

Antibodies used	Abcam ab122932 R&D Systems AF3369; R&D Systems A5441; Abcam ab22569; Santa Cruz sc-365823; Sigma SAB4200080; Diagenode C15200203-100; Millipore 07-473, Abcam b8895a; Abcam ab4729
Validation	WE followed manufactoral protocols.

Eukaryotic cell lines

Policy information about cell lines and Sex and Gender in Research

Cell line source(s)	mouse E14 Thermo Fisher.
Authentication	Cells were authenticated by sequencing.
Mycoplasma contamination	Cells were tested for mycoplasma and were mycoplasma free.
Commonly misidentified lines (See ICLAC register)	does not apply

ChIP-seq

Data deposition

☒ Confirm that both raw and final processed data have been deposited in a public database such as GEO.

☒ Confirm that you have deposited or provided access to graph files (e.g. BED files) for the called peaks.

Data access links *May remain private before publication.*	To review GEO accession GSE168415 go to<br>https://www.ncbi.nlm.nih.gov/geo/query/acc.cgi?acc=GSE168415<br>Enter token qhmrsyggntwrnkv into the box.
Files in database submission	H3K27ac ; H3K4me1; H3K4me3 and Input DNA in EpiLCs and ME.
Genome browser session (e.g. UCSC)	*Provide a link to an anonymized genome browser session for "Initial submission" and "Revised version" documents only, to enable peer review. Write "no longer applicable" for "Final submission" documents.*

Methodology

Replicates	At least one replicate for each condition.
Sequencing depth	Single read, 76 bp. Number of sequenced reads: 20685440 (H3K27ac in EpiLCs); 29531696 (h3K4m1 EpiLCs); 22014065 (H3K4me3 EpilCs); 20673711 (input EpioLCs); 29117898 (H327ac ME); 22713350 (H3K4me1 ME); 26492699 (H3K4me3 ME); 22491775 (input ME).
Antibodies	H3K4me3 07-473 Millipore<br>H3K4me1 ab8895 Abcam<br>H3K27ac ab4729 Abcam
Peak calling parameters	Peaks were called using MACS v2.1.1, with minimum FDR set to 0.05
Data quality	All peaks have FDR <= 5%. >50% peaks have fold-enrichment >=5.
Software	Mapping was performed using Bowtie v2.3.4.1 (options: -q --local). Duplicated alignments (identified by Picard MarkDuplicates - v2.21) and multi-mapping reads were excluded using SAMtools v 1.9. Peak calling was performed using MACS v2.1.1, with FDR cut-off at 0.05 (options: -q 0.05).
